# Context shapes action understanding in social interactions

**DOI:** 10.1016/j.isci.2026.117796

**Published:** 2026-10-09

**Authors:** Johannes Keck, Kazuyuki Sato, Rouwen Cañal-Bruland

**Affiliations:** 1 Department for the Psychology of Human Movement and Sport, Friedrich Schiller University, Jena, Germany

**Keywords:** kinematic coding, intention, social interaction, adaptive mentalization, anticipation, rock-paper-scissors, decision-making, reinforcement learning, context

## Abstract

In social interactions, individuals anticipate what others are about to do next, enabling smooth social interactions and coexistence in daily life. Here, we examined how kinematic and contextual cues are weighted and integrated to predict an interactor’s actions during dynamic, real-time competitive interactions. We analyzed 76 participants (38 dyads) freely playing 300 games of rock-paper-scissors each while tracking their kinematics using motion capture. Although movement kinematics consistently conveyed intention-related information, successful action selection depended primarily on how this information influenced predictions. Reinforcement-learning based contextual inference emerged as the strongest predictor of single-trial success and modulated the degree to which movement-derived predictions guided behavior. When contextual and movement-derived information afforded different actions, prioritizing contextual inference consistently outperformed movement-derived predictions. Thus, successful social action understanding depends not on maximizing sensitivity to movement cues, but on adaptively arbitrating between movement-derived and outcome-based contextual information during ongoing social interaction.

## Highlights

• Movements convey intention-related information in social real-time interactions
• Reinforcement-learning inference is the strongest predictor of single-trial success
• Contextual inference regulates when movement-derived predictions guide behavior
• Contextual predictions outperform kinematic predictions when these are in conflict

## Graphical abstract

**Figure:**
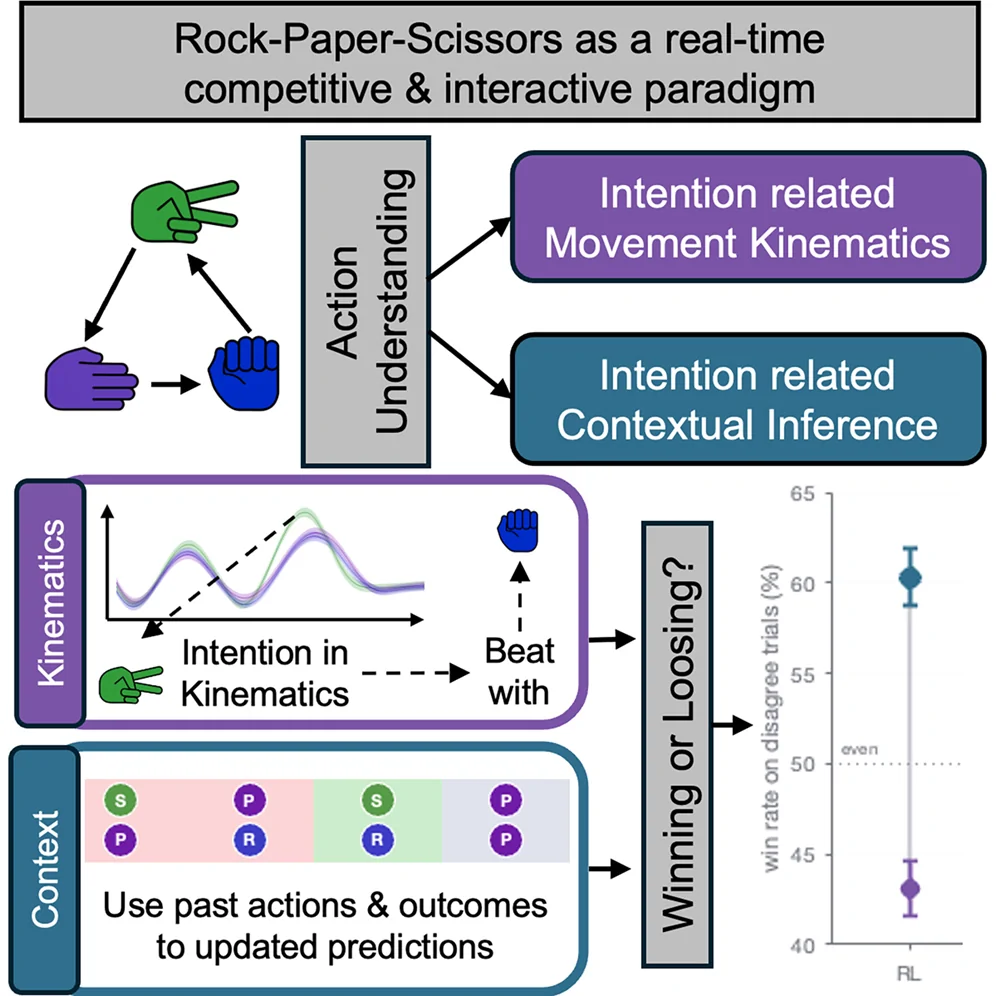

## Teaser

Social sciences; Psychology

## Introduction

In everyday social interactions individuals anticipate what others are about to do next. This capacity ultimately enables smooth social coexistence among humans. To anticipate, observing and reading movements serves as a window into other peoples’ intentions. Subtle variations in observed movements carry important information about (i.e., encode) different intentions or goals of action. For instance, if someone is grasping a bottle positioned in the middle of a roundtable that is surrounded by several people, one might question themselves whether the observed person is grasping for and lifting the bottle to pour water into their own or another person’s glass. Although the initial grasping movement may appear almost identical between those two action alternatives, fine-grained movement variations such as grip height, wrist orientation, velocity, movement trajectory or timing patterns do, in fact, allow to readout the true intended action.^1^^,^^2^^,^^3^^,^^4^^,^^5^^,^^6^^,^^7^

Moment-to-moment variations in movement offer a rich embodied signal of unfolding mental states and reflect internal cognitive dynamics such as decision confidence and even spontaneous changes of mind.^8^^,^^9^^,^^10^^,^^11^^,^^12^ It follows that subtle variations encoded in movements (i.e., kinematics) do allow to anticipate (i.e., predict) action intentions well above chance level. In fact, the capability to readout encoded movement patterns during social interactions has been identified as a human skill that based on experience and development can reach high levels of context-specific expertise.^1^^,^^2^^,^^7^^,^^12^^,^^13^

Regarding context-specificity, it is important to note that action observation and actions—just like the bottle grasp illustrated before—do seldom occur in isolation. In fact, more often than not actions and interactions take place in specific social contexts. Moreover, these social contexts tend to vary. That is, the same reaching movement described before may afford an even different interpretation if a little, playful child was sitting close to the bottle. In such a situation, next to pouring a drink for oneself or for someone else, the observed individual may actually be grasping the bottle with the intention to safeguard the bottle from the child by putting it out of reach. Hence, to anticipate other peoples’ actions in social interactions, both action intentions encoded in movements as well as social context may be informative.

The example of observing someone reaching for a bottle in various social contexts serves to illustrate that reading out truthful action intentions from movements is challenging, but possible.^1^^,^^2^^,^^5^^,^^7^ Yet, anticipating action intentions becomes far more challenging when faced with disguised or deceitful action intentions. Such strategies are commonplace in competitive social situations.^14^ For instance, in sports competitive actions such as penalty situations are abundant. There is a wealth of studies showing that in such competitive scenarios, individuals oftentimes disguise or deceive their true intentions to increase uncertainty about their actions. They do so by deliberately changing their kinematic information into kinematic *dis*information.^15^^,^^16^^,^^17^^,^^18^ However, when kinematic information is ambiguous or strategically manipulated, observers may rely more heavily on contextual cues to infer others’ intentions.^3^^,^^19^ In such cases, predictions can be guided by knowledge of past behavior, expectations about likely action tendencies (e.g., a penalty taker’s preferred kicking direction), or recursive belief-based reasoning in strategically interdependent settings.^20^^,^^21^^,^^22^ Additionally, outcome-dependent adjustments—such as a bias to repeat successful actions—can shape anticipatory judgments.^23^^,^^24^ Research hence seems to suggest that strategic regularities can be inferred from contextual information, particularly when kinematic cues are unreliable.^3^

Taken together, when aiming to anticipate what others are about to do next in social interactions insight into an observed person’s intention might emerge both from processes that integrate prior contextual information—such as past behavior, situational constraints, or task structure—together with current sensory evidence, including observable movement kinematics.^25^^,^^26^^,^^27^ In this vein, initial evidence seems to suggest that prior expectations are predominantly relied on when the kinematic encoding and, in particular, readout are unreliable, whereas prior expectations are relied on less the more reliable the kinematic information.^3^ This initial evidence, however, has severe limitations, as most studies have not truly addressed real-time, dynamic social encounters between actors, as they typically study kinematic observable cues and contextual inference in isolation.^2^^,^^7^^,^^11^^,^^24^^,^^28^^,^^29^^,^^30^^,^^31^^,^^32^^,^^33^ Moreover, while kinematic information is commonly assessed at the single-trial level using two-alternative forced-choice tasks in non-competitive settings, contextual inference is often examined only at the participant level without accounting for kinematic cues.^2^^,^^7^^,^^11^^,^^28^^,^^34^ Such designs fail to capture real-life social interactions, in which observers continually respond to each other in real-time.^35^^,^^36^ It is therefore that our understanding of how kinematic and contextual cues are integrated during dynamic, real-time social interactions and how conflicts between these different sources of information are resolved remains fundamentally limited.

To improve our understanding dynamic, real-time social interactions and to address the aforementioned lacuna, in the present study we examined how humans continuously integrate kinematic and contextual information during social interactions while playing the game rock-paper-scissors (RPS).^37^ Playing iterative games of RPS served as an ideal paradigm as it entails both a competitive scenario and more than two action possibilities. RPS is particularly suitable as it offers a unique, well-known task in which visually observable movement kinematics unfold in real time alongside rich contextual information derived from the game’s structure and history of play. As a zero-sum game with a non-transitive payoff structure—rock beats scissors, scissors beats paper, and paper beats rock (see Figure 1)—each player’s gain directly corresponds to the other’s loss.^30^^,^^38^^,^^39^ Although optimal play predicts random choice distributions, human players reliably deviate from this strategy, exhibiting systematic biases and sequential patterns that are exploitable by algorithmic agents.^34^^,^^40^^,^^41^^,^^42^ These regularities make iterated RPS a powerful model system for studying interactive anticipation in fast-paced competitive interactions with multiple possible actions^11^^,^^32^^,^^35^ (see Figure 1A). Our specific aims were 3-fold: we examined (1) whether subtle kinematic features allow observers to anticipate an opponent’s action before it unfolds, (2) whether strategic regularities can be inferred from contextual information, and (3) how humans resolve conflicts between these information sources.

**Figure 1:**
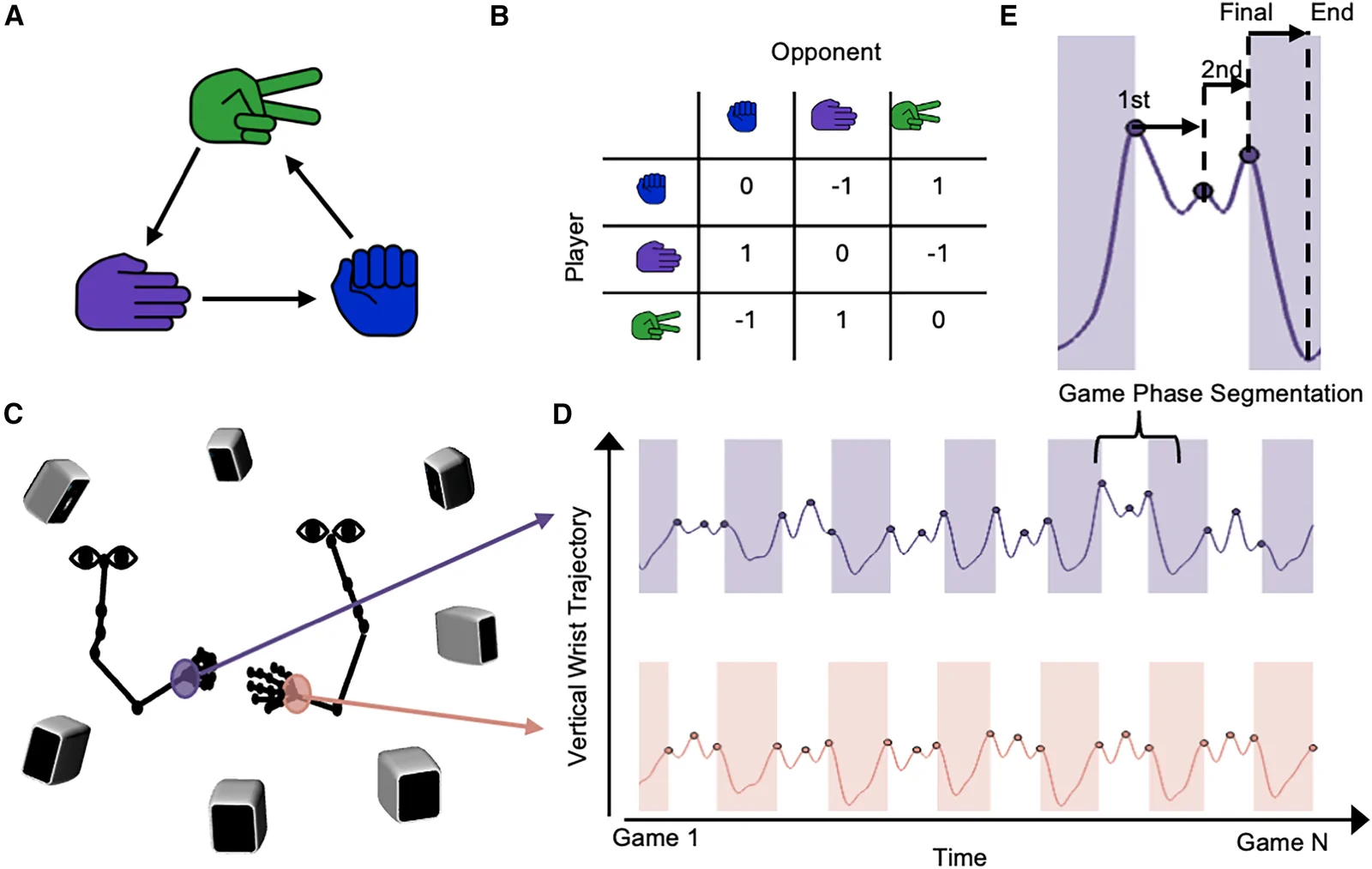
The RPS game and game-phase segmentation (A) Cyclic dominance among the three actions. (B) Payoff matrix. Each element shows the payoff for the player’s action against the opponent (1 = win, 0 = draw, −1 = loss). (C) Motion capture of wrist kinematics. (D) Example of consecutive RPS games within a dyad. Wrist trajectories depict upward and downward swings; shaded area indicates the game-evaluation phase. (E) Game-phase segmentation procedure within a single game.

To test these questions, participants performed a competitive, dyadic RPS task while being equipped with full-body motion capture suits (see Figure 1C and Video S1). On each trial (game), two participants freely selected an action (see Figure 1B) while performing the characteristic up-down wrist swing, yielding naturally generated kinematics and outcomes (win/draw/loss) across consecutive games. We first quantified how much intention information was *encoded* in single-trial wrist kinematics (see Figure 1D) by fitting a multinomial classifier that mapped phase-specific movement features to the played action (rock, paper, scissors). We then quantified how effectively this kinematic information was likely *readout* by opponents by asking whether their actual choice matched the optimal countermove given the kinematics on a given trial, thus providing an implicit, single-trial measure of likely kinematic readout. Notably, likely readout is not a direct measure of perceptual readout but an approximation derived from players’ choices. It quantifies the extent to which a player’s selected action is consistent with the action that would be optimal given the decoder’s kinematic evidence. In the present RPS paradigm, this inference assumes that players aim to maximize their probability of winning by selecting the action that defeats the opponent, while acknowledging that other unobserved factors may also influence choice. Finally, we modeled contextual inference from each player’s recent action history using a set of competing computational models spanning two conceptually distinct families: action-based models, which predict choices from sequential regularities in previous actions (e.g., Markov, cognitive hierarchy assessment [CHASE]), and outcome-based models, which adapt behavior according to previous wins and losses (e.g., win-stay/lose-shift, reinforcement learning [RL]).^11^^,^^23^^,^^41^^,^^43^^,^^44^^,^^45^ This distinction allowed us to test whether playing successfully is better explained by exploiting sequential action patterns or by reward-driven adaptation. Model-derived predictions were evaluated together with kinematic readout in hierarchical single-trial analyses to quantify their independent and competing contributions to moment-to-moment performance (for details, see STAR Methods).

## Results

### Successful kinematic readout differentiates winning from losing

The encoding model estimates how much intention information is embedded in the movement kinematics (see Table 1 for details on the calculation of the movement kinematics) and could, in principle, be extracted by an ideal observer. Across all trials, the classifier decoded the opponent’s intended action with an accuracy of 47.6% (95% CI [46.9%, 48.3%]), significantly exceeding chance level (33.3%, *p* < 0.001). To examine whether encoding differed between winning and losing trials or across movement phases, we fitted a hierarchical binomial generalized linear mixed-effects model including outcome (win/loss), movement phase (first, second, and final swing), and their interaction, with random intercepts for participant and dyad. Neither outcome (χ^2^(1) = 0.03, *p* = 0.874), movement phase (χ^2^(2) = 0.39, *p* = 0.821), nor their interaction (χ^2^(2) = 0.59, *p* = 0.743) significantly affected encoding accuracy. Consistent with these findings, encoding accuracy remained significantly above chance for every movement phase and outcome combination after Holm correction (all *p* < 0.001; Figure 2A).

**Table 1:** Movement features

Movement feature	
Feature	Definition
**Trajectory Features**	

Peak shakiness	positional variability of the wrist in 3D
Trajectory skewness	asymmetry in motion path

**Temporal Features**	

Duration (s)	total time of movement interval
Time to peak velocity	time until peak velocity occurs
Early velocity ratio	how much contributes the early part of the movement to the total velocity

**Kinematic Features**	

Peak velocity (cm/s)	maximal wrist velocity within each game phase
Peak acceleration (cm/s^2^)	maximal wrist acceleration within each game phase
Mean velocity	average velocity within each game phase
Median velocity	–
Velocity STD	standard deviation of velocity
Velocity entropy	measure of unpredictability of velocity changes

Definition of movement features for classification of action pose.

**Figure 2:**
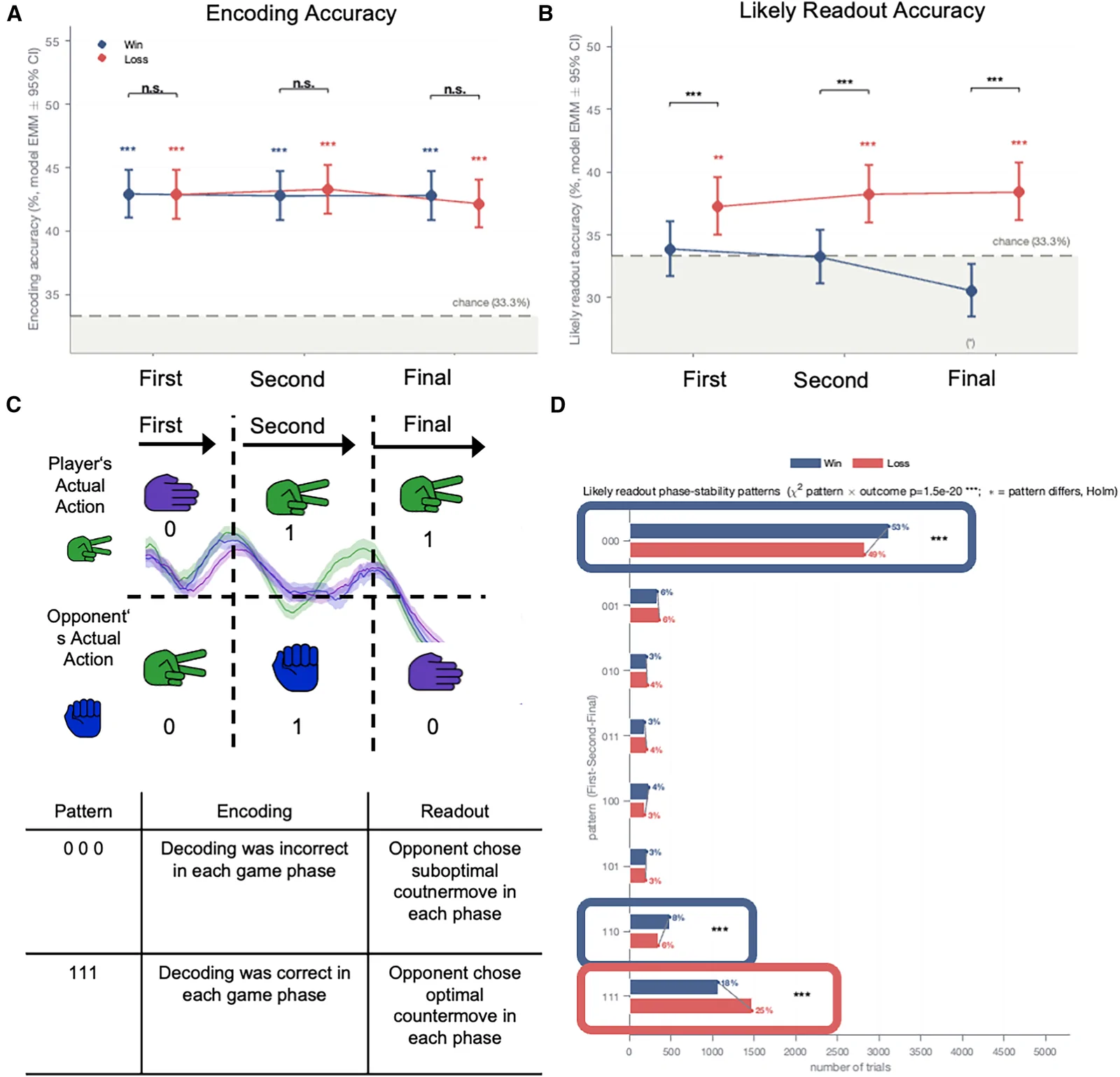
Kinematic inference of RPS actions (A) Kinematic encoding. Encoding accuracy was significantly above chance in all three game phases but did not differ between winning and losing trials nor across phases (estimated marginal means ± 95% CI). (B) Kinematic readout. Readout accuracy diverged by outcome and phase (Outcome × Phase interaction, *p* < 0.05). For loosing trials, it remained above chance but for winning trials it declined in the final phases (estimated marginal means ±95% CI). (C) Encoding and readout stability. Schematic illustration of pattern stability across the three game phases. (D) Readout pattern stability. Proportion of stable readout for winning and losing trials.

To assess whether participants’ choices aligned with the information available in the opponent’s movement, we quantified likely kinematic readout (see Figure 3 for the operationalisation ot the likely kinematic readout used in this paper) as the probability that the participant’s response was consistent with the action implied by the decoded kinematics. Overall likely readout reached 39.2% (95% CI [38.5%, 39.9%]), significantly exceeding chance level (33.3%, *p* < 0.001). Hierarchical mixed-effects analysis revealed a robust main effect of outcome (χ^2^(1) = 101.52, *p* < 0.001), no main effect of movement phase (χ^2^(2) = 3.91, *p* = 0.142), and a significant outcome × phase interaction (χ^2^(2) = 10.83, *p* = 0.004). Post hoc comparisons showed that likely readout did not exceed chance on winning trials in any movement phase (first: 34.0%, second: 33.7%, final: 31.1%; all Holm-corrected *p* ≥ 0.108), whereas it was significantly above chance throughout losing trials (first: 37.6%, second: 38.3%, final: 38.6%; all Holm-corrected *p* < 0.001). Moreover, likely readout was significantly higher during losing than winning trials in each movement phase (all Bonferroni-corrected *p* < 0.001), with the largest difference observed during the final swing (Figure 2B). This dissociation indicates that intention information was encoded equally well in the movement kinematics regardless of game outcome, whereas behavioral alignment with the likely kinematic readout was selectively associated with unsuccessful decisions.

**Figure 3:**
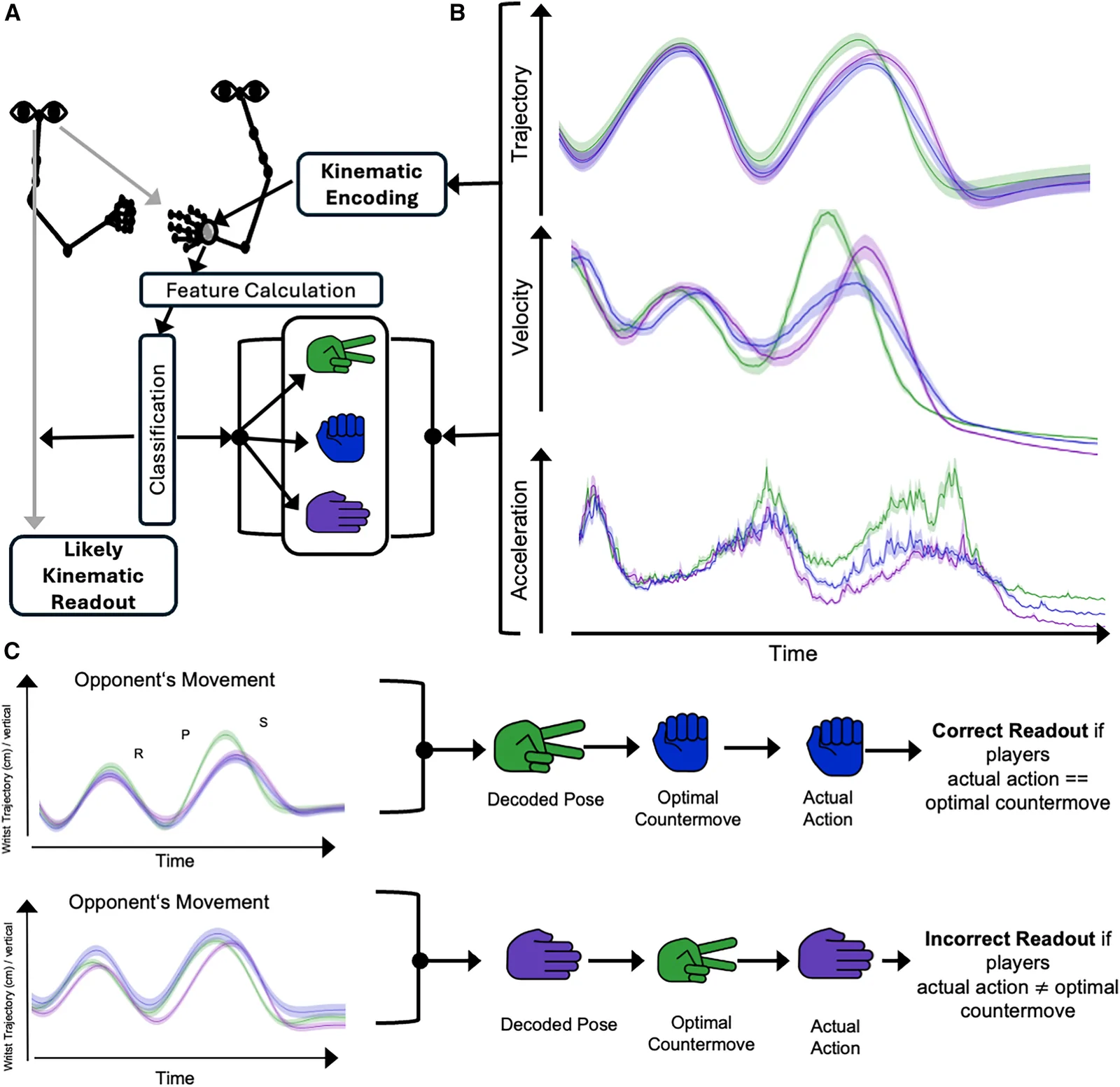
Kinematic inference of intentions in RPS (A) Kinematic inference pipeline within a single game. The player observes the opponent’s movement and selects the corresponding action. (B) Kinematic encoding. Exemplary movement features from one participant that were used for classification. For feature definition, see Table 1. (C) Likely kinematic readout. Definition of correct and incorrect readout based on the opponent’s kinematic encoding.

Descriptive statistics for encoding and likely readout across game phases and sessions are summarized in Table S1, along with participant-level averages for encoding (Table S2) and likely readout (Table S3).

To further assess how intention-related information unfolded and was utilized over time, we analyzed game phase-specific stability scores to determine whether encoded and interpreted information was consistently maintained or changed throughout the course of the game (see Figure 2D). Consistent with the game-phase-wise decoding analyses, all encoding measures—including weighted posterior strength, binary decoding accuracy, and phase stability—did not differ between winning and losing trials (all *p* > 0.63). Likewise, the distribution of encoding stability patterns was comparable between outcomes (χ^2^, *p* = 0.743), indicating that the temporal evolution of encoded intention information was unaffected by game outcome.

In contrast, all measures of likely kinematic readout consistently differentiated winning from losing trials. Weighted likely readout strength (32.6% vs. 38.4%; *t*(11670) = −7.67, *p* < 0.001, *d* = −0.14), binary likely readout (33.1% vs. 38.4%; *t*(11670) = −6.90, *p* < 0.001, *d* = −0.13), and likely readout stability (82.1% vs. 83.9%; *t*(11678) = −3.25, *p* = 0.001, *d* = −0.06) were all significantly higher during losing than winning trials. These findings were confirmed by hierarchical mixed-effects models (all *p* < 0.001).

The corresponding stability patterns mirrored these results. Whereas encoding patterns occurred with comparable frequencies for winning and losing trials, likely readout patterns differed significantly (χ^2^, *p* < 0.001). Winning trials were characterized by a higher consistent absent likely readout (pattern **000**) and intermediate readout patterns (e.g., **100**, **110**) especially in the final phase of the game, whereas losing trials were significantly more present in consistent likely readout pattern (**111**). Together, these findings indicate that intention information was encoded with comparable temporal stability regardless of outcome, whereas consistently following the likely kinematic readout across movement phases was associated with unsuccessful decisions.

At first glance, the single-trial pattern may seem counterintuitive, as more accurate likely kinematic readout would be expected to support optimal counter-moves. However, in fast-paced competitive interactions with a multitude of action outcomes, kinematic cues are noisier (see Figure 2A) or ambiguous. Hence, under these conditions, observers may benefit from complementing kinematic information with contextual cues to guide action selection.

### Contextual and kinematic predictors of single-trial winning

The kinematic analyses raise the question of whether successful action understanding in competitive settings depends less on kinematic readout alone and more on the ability to infer strategic regularities from contextual information, we modeled single-trial win probability using the posterior probabilities of four competing contextual models (Markov, Win Stay Loose Shift in the following WSLS, RL, and CHASE) together with kinematic encoding strength and likely kinematic readout strength in a hierarchical mixed-effects logistic regression (Figure 4A).

**Figure 4:**
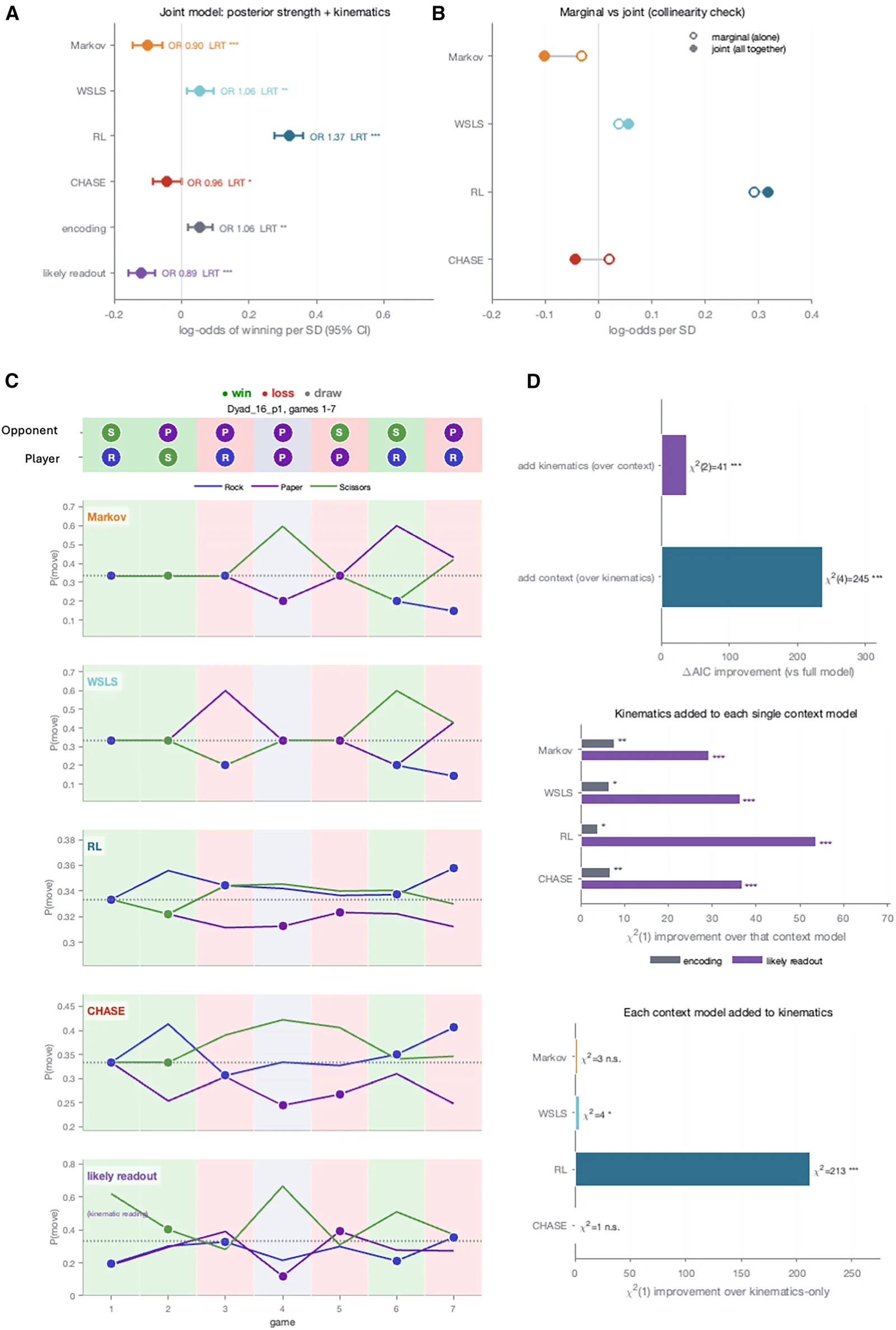
Kinematic and contextual contribution to single-trial-winning (A) Joint-model estimates (log-odds per SD ± 95% CI). RL was the strongest positive predictor (QR = 1.37). (B) Marginal (alone) vs. joint (kinematic + context) estimates. (C) Example action posteriors over seven consecutive games of one dyad. Played moves, outcome, and action choice. Schematic illustration of pattern stability across the three game phases. (D) Nested likelihood-ratio tests: both blocks added unique fit (kinematics χ^2^(2) = 41; context χ^2^(4) = 245); the readout contributed over every context model, whereas the context effect was carried almost entirely by RL (χ^2^(1) = 213). (∗*p* < 0.05, ∗∗*p* < 0.01, ∗∗∗*p* < 0.001).

Among the contextual models, RL showed the strongest positive association with winning. Trials on which the RL model assigned a higher posterior probability to the participant’s chosen action were significantly more likely to result in a win (β = 0.318, OR = 1.37, 95% CI [1.32, 1.43], *p* < 0.001). A smaller positive effect was observed for the WSLS model (β = 0.056, OR = 1.06, *p* = 0.007). In contrast, greater posterior probability under the first-order Markov model predicted a reduced probability of winning (β = −0.102, OR = 0.90, *p* < 0.001), indicating that reliance on sequential action regularities was associated with more exploitable behavior. Posterior probability under the recursive CHASE model showed only a weak negative association with winning (β = −0.044, OR = 0.96, *p* = 0.044).

The kinematic predictors revealed a complementary dissociation. Greater encoding strength was associated with a modest increase in winning probability (β = 0.057, OR = 1.06, *p* = 0.003), whereas greater likely kinematic readout strength predicted a significantly lower probability of winning (β = −0.118, OR = 0.89, *p* < 0.001). Thus, although richer intention information embedded in the movement was beneficial, stronger behavioral alignment with the likely kinematic readout was associated with poorer performance.

These findings proved robust across complementary analyses including draws (see Figure S5). Marginal models yielded highly similar estimates and collinearity among contextual predictors was low (all Variance Inflation Factors (VIF) ≤ 1.35; see Figure S4). Together, these results identify RL as the contextual process most strongly associated with successful performance, whereas reliance on action-based sequential regularities and likely kinematic readout was associated with reduced success.

### Integration of contextual and kinematic information

To determine whether kinematic and contextual information contributed independently to successful performance or were integrated during action selection, we extended the single-trial mixed-effects model by including interactions between the two kinematic predictors (encoding strength and likely kinematic readout strength) and each contextual model (Figure 4D). Both information sources contributed unique predictive information. Relative to a context-only model, adding the two kinematic predictors significantly improved predictive model fit (likelihood-ratio test: χ^2^(2) = 33.35, *p* < 0.001; Figure 4D). Conversely, adding the four contextual predictors to a kinematics-only model yielded a substantially larger improvement in predictive model fit (χ^2^(4) = 244.65, *p* < 0.001; Figure 4D), indicating that contextual information explained a greater proportion of trial-to-trial variation in winning, while kinematic information still provided significant complementary predictive value.

Beyond these additive contributions, the interaction model significantly improved predictive model fit relative to the main-effects model (χ^2^(8) = 28.68, *p* < 0.001; Figure 5A), demonstrating that the relationship between kinematic information and successful performance depended on the concurrent contextual state. Examination of the individual interaction terms revealed that this effect was driven specifically by RL. After correction for multiple comparisons, only the interaction between reinforcement-learning posterior strength and likely kinematic readout strength remained significant (β = −0.070, *p* = 0.016, Holm-corrected; Figure 5A), whereas none of the interactions involving kinematic encoding or the remaining contextual models reached significance.

**Figure 5:**
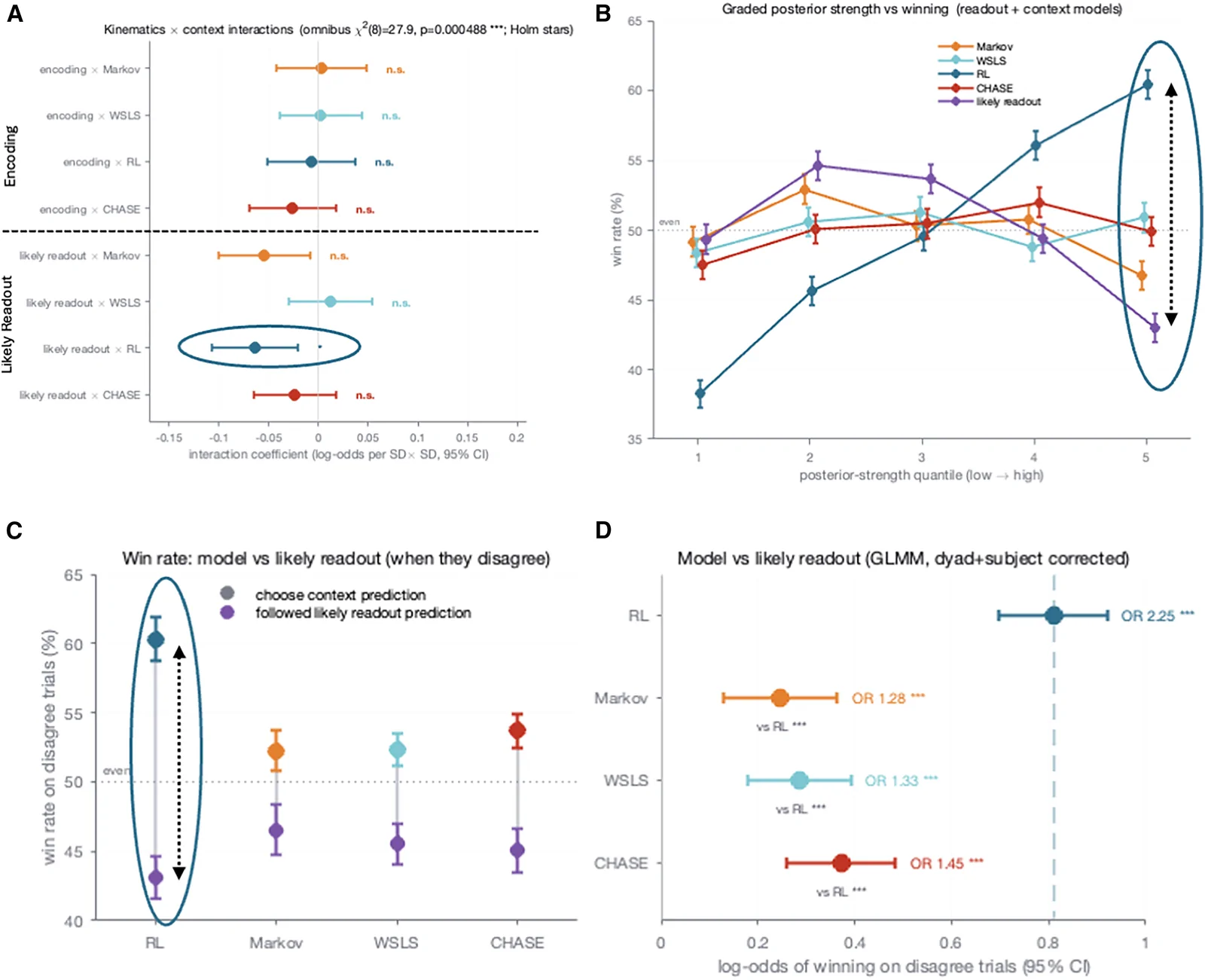
Kinematic and contextual interaction and conflict to single-trial-winning (A) Kinematics × context interaction terms (log-odds per SD ± 95% CI). Interaction was mainly driven by a negative likely readout x RL term, indicating that the behavioral influence of the likely kinematic readout decreased as RL evidence increased. (B) Graded effects of posterior strength (probability). RL posterior showed a linear increase with winning, whereas readout showed a negative relationship. Markov posterior likewise decreased with winning. (C) Context-kinematic conflict. Across all contextual models, following the contextual model increased winning probabilities compared to the. Likely kinematic readout. (D) Model comparison on disagreement trials. Mixed-effects logistic regression odds ratios comparing context-guided vs. readout-guided decisions. Odds ratios greater than one indicate that following the context increased the odds of winning relative to following the likely kinematic readout. RL showed the strongest advantage, significantly exceeding the benefits of Markov, WSLS, and CHASE.

The negative interaction coefficient indicates that the behavioral impact of the likely kinematic readout became increasingly detrimental as reinforcement-learning evidence for the chosen action increased. In contrast, the contribution of kinematic encoding remained additive across contextual states. Thus, outcome-based contextual information selectively modulated the behavioral use of movement-derived predictions rather than the availability of kinematic information itself.

### Contextual inference outperforms kinematic signals in competitive interactions

To address how participants resolved conflicts between kinematic cues and contextual information during dynamic competitive interaction, we restricted the analysis to decisive trials on which the contextual model and the likely kinematic readout recommended different actions (Figure 5C). These disagreement trials provide a direct test of how participants resolve conflicts between contextual and kinematic information, because successful performance can only be attributed to one of the two competing information sources.

Although all contextual models outperformed the likely kinematic readout under conditions of disagreement, their behavioral benefits differed substantially. The strongest effect was observed for the reinforcement-learning model (β = 0.848, OR = 2.33, 95% CI [2.09, 2.61], *p* < 0.001), indicating that selecting the RL-recommended action more than doubled the odds of winning relative to following the likely kinematic readout. Descriptively, participants won 60.6% of disagreement trials when following the RL recommendation, compared with only 42.7% when following the likely kinematic readout.

The remaining contextual models also significantly outperformed the likely kinematic readout, although with considerably smaller effects (see Figure 5D). Following the CHASE recommendation increased the probability of winning (OR = 1.51, *p* < 0.001), as did WSLS (OR = 1.38, *p* < 0.001) and the first-order Markov model (OR = 1.34, *p* < 0.001). Thus, even relatively simple contextual models provided greater behavioral benefit than immediate reliance on movement-derived information whenever the two sources conflicted.

Direct comparison of the contextual models further demonstrated the superiority of RL. Across participants, following the RL recommendation yielded a 17.9 percentage point increase in winning probability relative to the likely kinematic readout, significantly exceeding the advantages of Markov (6.1 percentage points), WSLS (7.5 percentage points), and CHASE (9.3 percentage points) (all Holm-corrected *p* < 0.001).

Together, these findings demonstrate that successful action selection during competitive interaction is best supported by reinforcement-learning-based contextual inference rather than by immediate behavioral alignment with the likely kinematic readout. When contextual expectations and movement-derived information diverged, prioritizing reinforcement-learning predictions consistently yielded the greatest behavioral advantage, whereas reliance on kinematic readout alone was associated with substantially lower success.

In summary, these results show that although bodily movements convey intention-related information, successful performance in competitive interaction depends on how kinematic cues are weighted against contextual expectations. Winning the game increasingly relied on contextual inference, indicating that effective action understanding emerges from resolving conflicts between information sources rather than from kinematic evidence alone.

## Discussion

Understanding what shapes successful social interactions is central to the fields of psychology, cognitive science, neuroscience, and economics and crucially important for both cooperative and competitive interactions. To improve our understanding of dynamic, real-time social interactions, in the present study we examined when and how kinematic and contextual cues are weighted and integrated to predict what another person will do next. Although kinematic information has been established as a key source for predicting others’ behaviors,^1^^,^^2^^,^^3^^,^^7^ most experimental approaches conceptualize this process from a third-person perspective, neglecting the role of the observer as an active part within the interaction.^35^^,^^36^ We therefore studied dynamic, real-time social interactions by inviting dyads of participants to play hundreds of games of RPS. Our findings provide first evidence, in a genuinely interactive and dynamically updated competitive setting, that even when kinematic information is available, its utilization is shaped and informed by continuously emerging contextual information. When kinematic and contextual cues disagree, successful interactors increasingly weight contextual inference over bodily signals to resolve informational conflicts.

In real-time competitive interactions, kinematic information is indeed available, as demonstrated by our encoding and classification results. However, the extent to which this information is successfully read out—and translated into adaptive behavior—varies as a function of outcome. Our single-trial readout analysis revealed that stronger utilization of kinematically encoded information was associated with a higher probability of losing the game. At first glance, this finding appears counterintuitive, as previous work would predict that more effective readout of kinematic cues should increase the likelihood of successful action prediction and, consequently, winning.^1^ Instead, our results indicate that in contexts characterized by multiple possible action outcomes, and actors not necessarily aiming at displaying their true action intentions, successful action understanding does not depend on maximal reliance on kinematic information, but on the relative weighting of the available information sources. In such cases, observers may generate several competing kinematic predictions, reducing accuracy and reliability despite high perceptual sensitivity.^46^

When anticipating an opponent’s next move during competitive interaction, movement-derived and contextual information did not contribute equally to successful action selection. Rather, movement kinematics provided informative, above-chance hypotheses about an opponent’s forthcoming action, but successful behavior did not arise from maximizing reliance on these movement-derived predictions. Instead, outcome-dependent contextual information—particularly RL—was associated with whether movement-derived information guided behavior. This conclusion is supported by three converging findings: (1) reinforcement-learning posterior strength was the strongest positive predictor of winning, whereas stronger likely kinematic readout predicted unsuccessful decisions; (2) reinforcement learning selectively moderated the behavioral impact of likely kinematic readout, indicating that reliance on movement-derived predictions became increasingly maladaptive as outcome-based contextual evidence accumulated; and (3) whenever contextual and movement-derived information afforded different actions, following the contextual recommendation consistently outperformed following the likely kinematic readout, with reinforcement learning providing the largest behavioral advantage. Together, these findings suggest that successful action anticipation depends less on maximizing sensitivity to movement kinematics rather than on adaptively regulating when movement-derived evidence should influence behavior.

These findings also help clarify the role of movement-derived information during competitive interaction. Previous work by Koul and colleagues^3^ showed that highly informative and distinguishable movements exert a stronger influence on information uptake than less informative movements. We extend this work by showing that, in competitive and time-pressured interactions, the behavioral value of movement-derived information depends not only on how much information is available but also on whether contextual evidence supports acting upon it. Thus, the utility of likely kinematic readout appears to depend on the broader contextual state rather than being fixed. One important contrast to previous kinematic decoding studies is that intention attribution is typically assessed using explicit perceptual reports. Here, intention attribution was instead inferred from the observer’s own action, such that selecting a counter-move (e.g., rock) indexed the inferred intention (e.g., scissors) conveyed by the opponent’s movement. This action-based measure is consistent with evidence that neural signatures of action prediction emerge most strongly during unconstrained interactive play rather than under explicit prediction instructions in RPS.^31^

More broadly, the present findings are consistent with previous computational work on repeated RPS. Across studies, outcome-dependent learning and other low-order sequential mechanisms account for much of human behavior, whereas evidence for stable higher-order recursive reasoning remains comparatively limited.^11^^,^^23^^,^^39^^,^^41^^,^^43^^,^^44^ Consistent with this literature, reinforcement-learning-based contextual inference outperformed first-order Markov, WSLS, and the recursive CHASE model in predicting single-trial success. Likewise, Guennouni and Speekenbrink^45^ reported that RL provided the best overall account of repeated zero-sum game behavior, whereas higher-order belief models primarily conferred advantages when knowledge had to be transferred across games rather than during ongoing interaction. Together, these findings suggest that adaptive behavior during continuous competitive interaction depends primarily on learning from accumulated outcomes rather than increasingly sophisticated recursive opponent modeling or relying solely on action-based predictions. In this regard, the present study extends this literature by jointly considering movement-derived and contextual information. Whereas previous computational studies have largely compared competing contextual models, we quantified the complementary and interacting contributions of movement-derived and contextual information to successful action selection within the same trial-level framework. Movement-derived information provided predictive value beyond contextual inference, contextual information explained additional trial-by-trial variation beyond movement-derived information, and reinforcement-learning signals selectively moderated the behavioral impact of likely kinematic readout without altering the intention-related information expressed in the movement itself. Moreover, when contextual and movement-derived information afforded different actions, following contextual predictions was associated with higher winning probabilities than following the likely kinematic readout. Together, these findings suggest that successful action understanding depends not only on extracting information from movement, but also on flexibly weighting movement-derived and contextual evidence according to their behavioral relevance. This interpretation is also consistent with previous work showing that contextual priors influence action prediction even when informative kinematic information is available.^29^ However, Bianco and colleagues examined static contextual priors acquired outside the interaction. By contrast, the contextual beliefs examined here were continuously updated through repeated interaction with an adapting opponent, allowing accumulated outcome information to influence when movement-derived information was translated into action.

In such interactive settings, opponents may strategically manipulate kinematics through deceptive or ambiguous movements. Under these conditions, the reliability of kinematic information decreases, and successful action understanding increasingly depends on contextual inference about an opponent’s strategy and likely action choice. Evidence that contextual information is represented on a neural level in RPS comes from hyperscanning work showing that neural activity during decision formation encodes information about previous actions of oneself and the opponent.^32^ Notably, this trial-history-based contextual encoding was primarily expressed in overall match losers, leading the authors to suggest that reliance on immediate past outcomes may hinder performance. This finding led to the suggestion that maintaining information about recent outcomes may impair optimal performance by increasing behavioral predictability. Our results provide a computational refinement of this interpretation. Rather than demonstrating that contextual information is generally maladaptive, they suggest that different forms of contextual information have markedly different behavioral consequences. Whereas first-order sequential dependencies were associated with lower winning probability, reinforcement-learning-based contextual inference emerged as the strongest predictor of successful action selection. Thus, successful competitive behavior does not appear to depend on representing more contextual information but on representing the appropriate form of contextual information. Consistent with this distinction, evidence from non-interactive action prediction tasks in RPS shows increased mirror neuron activity when predictions align with observed outcomes, underlying the predictive capacity to understand the action of another.^33^ Moreover, the present study extends these neural findings by demonstrating that contextual representations do not operate in isolation. Instead, reinforcement-learning signals regulated the behavioral influence of movement-derived information, indicating that contextual information serves not merely to predict future actions but to determine when observable kinematics should guide behavior. Future neuroimaging work may therefore benefit from distinguishing neural representations associated with RL, simple sequential regularities, and movement-derived action predictions, rather than treating contextual information as a unitary construct.

Importantly, the present findings demonstrate that movement-derived and contextual information are neither redundant nor processed independently. Both sources provided complementary information about single-trial success, yet contextual information accounted for substantially more unique predictive variance than movement-derived information. Beyond these additive contributions, reinforcement-learning posterior strength selectively modulated the behavioral impact of likely kinematic readout, indicating that the utility of movement-derived predictions depended on the strength of accumulated outcome-based evidence. This interaction was further corroborated by the conflict analyses, which showed that whenever contextual and movement-derived information afforded different actions, prioritizing reinforcement-learning predictions consistently yielded superior performance. These findings extend previous evidence for continuous integration of contextual information in action understanding by showing that, in time-pressured competitive interactions with direct feedback, contextual inference actively regulates *when* kinematic information should guide behavior.^29^

Crucially, in genuinely interactive social interactions, the predictive validity of kinematic cues is not fixed, but depends on the contextual state in which that movement is interpreted. Although movement kinematics consistently contained intention-related information, successful plays did not benefit from maximizing behavioral alignment with the likely kinematic readout. Instead, movement-derived information contributed most effectively when evaluated in conjunction with dynamically updated outcome-based contextual evidence. This distinction between the availability of movement information and its behavioral utilization suggests that successful action understanding in competitive interaction depends on adaptive arbitration between multiple information sources rather than on maximizing sensitivity to any single cue.

The present findings demonstrate that successful action understanding in dynamic competitive, real-time, interactions depends on adaptive arbitration between movement-derived and contextual information rather than on either source alone. Although human movement consistently conveyed intention-related information, successful action selection was determined by how this information was behaviorally used. Outcome-based contextual inference—captured most strongly by RL—provided the dominant predictor of single-trial success, while movement-derived information contributed complementary predictive value. Importantly, contextual inference not only explained behavior independently of kinematics but was also associated with when movement-derived predictions guided actions. When contextual and kinematic information conflicted, prioritizing contextual inference consistently resulted in superior performance. Together, these findings suggest that social action understanding is best conceptualized not as maximizing sensitivity to movement cues, but as dynamically regulating their behavioral relevance according to accumulated contextual evidence. More broadly, our results identify adaptive arbitration between multiple sources of information as a candidate computational principle underlying human decision-making in uncertain, interactive environments.

### Limitations of the study

One limitation of this interpretation is that the present measure of likely readout constitutes an indirect behavioral proxy rather than a direct measure of perceptual action understanding. It infers the most likely kinematic readout from participants’ chosen responses under the assumption that players aim to maximize their probability of winning. Future studies could test this inference more directly by using immersive virtual reality with experimentally controlled avatar kinematics. For example, avatars could execute movements with systematically manipulated kinematic discriminability, allowing observers to either explicitly identify the intended RPS gesture or indicate which gesture they believe would optimally counter the observed movement. Comparing these explicit perceptual judgments with the inferred likely readout would provide a more direct validation of the behavioral proxy used in the present study.

## Resource availability

### Lead contact

Requests for further information and resources should be directed to and will be fulfilled by the lead contact, Johannes Keck (johannes.keck@uni-jena.de).

### Materials availability

This study did not generate new unique reagents.

### Data and code availability

• The data required to reproduce the results reported in this study are available on the Open Science Framework (OSF) at https://doi.org/10.17605/OSF.IO/89XS5. This repository contains the processed and pose-classified wrist-trajectory data used for the analysis.
• The full-body motion capture recordings are part of a larger ongoing research project funded by the Volkswagen Foundation (grant no. 9C251). These data are currently being used in additional experiments and will be made publicly available upon completion of the project and are available from the corresponding author upon reasonable request.
• The analysis code used in this study is not currently publicly available and will be made publicly available upon completion of the ongoing research project.
• Any additional information required to reanalyze the data reported in this paper is available from the corresponding author upon reasonable request.

## Acknowledgments

We sincerely appreciate the dedication of all the participants. We also extend our gratitude to Lukas Kanter and Georg Lindenberger for their assistance with data collection and participant recruitment as well as Felix Stieglbauer for providing feedback on the final draft. This project is supported by a Momentum research grant awarded by the 10.13039/501100001663VolkswagenStiftung to R.C.-B. (Az.: 9C251).

## Author contributions

J.K., conceptualization, methodology, data collection, data analysis, software, visualization, and writing – original draft; K.S., investigation, and writing – review and editing; R.C.-B., funding acquisition, conceptualization, methodology, supervision, and writing – review and editing.

## Declaration of interests

The authors declare no competing interests.

## STAR★Methods

### Key resources table

**Table:** 

REAGENT or RESOURCE	SOURCE	IDENTIFIER
**Deposited data**		

Data supporting main findings	This Paper	OSF Data: https://doi.org/10.17605/OSF.IO/89XS5.

**Software and algorithms**		

MATLAB (Version 2024b)	MathWorks Inc.	https://www.mathworks.com/products/matlab/; RRID:SCR_001622
JASP (Version 0.19.2)	JASP Team	https://jasp-stats.org/; RRID:SCR_015823
Motive motion capture software (Version 3.3)	OptiTrack (NaturalPoint, Inc.)	https://www.optitrack.com/software/motive

### Experimental model and subject participant details

#### Participants

We recruited 90 participants (45 females and 45 males; mean age = 24.1 years, SD = 2.6). The study was approved by the local ethics committee of the Friedrich-Schiller-University Jena (approval no. FSV 24/093), and all participants gave their informed written consent in accordance with the Declaration of Helsinki. Participants were paired into dyads (15 Female/Female; 15 Male/Male; 15 Female/Male).

### Method details

#### Task and procedure

Dyads were instructed to play repeated games of Rock-Paper-Scissors (see Figure 1A), with free action choices. Experimental blocks consisted of 10 blocks with 30 repeated games and a short break in between (mean game time = 1.4 s, SD = 0.4). Each participant was instructed to try to win as many games as possible, after each block participants received information about the current game statistics. Winner or Loser is defined by the game rules: Rock wins over Scissors; Paper wins over Rock and Scissors beats Paper (see Figures 1A and 1B). On average dyads took 30 minutes to complete the sequence of blocks. Participants’ hand and arm movement were recorded using a near-infrared camera motion capture system with 16 cameras, while wearing a motion capture suit with 57 reflective markers attached to the whole-body (frame rate, 240 Hz; OptiTrack Motion Capture System) (see Figure 1C). For the analysis only finger, wrist, forearm, upper arm and shoulder makers were used.

#### Pre-processing and hand pose classification

Prior to further analysis, hand and arm motion data were gap-filled using cubic spline interpolation, and erroneously labelled finger trajectories were visually inspected and corrected. Hand pose classification was performed in two stages.^47^ In the first stage, movement segmentation was conducted by smoothing the 3D wrist trajectory with a Gaussian filter (σ = 3). The vertical wrist displacement (see Figure 1D) was then analysed to segment the sequence into game phases: the first upward peak corresponded to *Rock* (First Swing), followed by a down-swing and second upward peak corresponding to *Paper* (Second Swing), and a subsequent down-swing and third peak corresponding to *Scissors* (Final Swing). The evaluation phase of the game ended (End of the Game) with the initiation of the next upward swing starting First Swing of the next game (see Figure 1E). In the second step, hand shape recognition was achieved from the 3D hand and finger trajectories from phase C to E. The distance between each fingertip (thumb, index, middle, ring, pinkie) to the wrist was calculated. Hand shape recognition was classified for each game and each participant. Hand shapes were classified according to their relative movement magnitudes. Movements with no or minimal movement were assigned to Rock, those with dominant activity in the index and middle finger to Scissors, and those with changes from index to pinkie as Paper.

Dyads that did not complete the full set of 300 games (*N* = 2) were excluded from further analyses. In addition, dyads were removed if, for one of the participants, finger and handshape trajectories could not be reliably reconstructed after pre-processing for at least 130 consecutive games (*N* = 5). Consequently, 38 of the initial 45 dyads were included in the final analysis (see supplemental information, Table S1 for exclusion criteria). All exclusions were performed after pre-processing and before data analysis.

#### Game outcome calculation

Classified handshapes were used to calculate the outcome of each game, given the rules: rock beats scissors, scissors beat paper and paper beats rock, while playing the same pose as the opponent was counted as a draw (see Figure 1B).

### Quantification and statistical analysis

#### Single-trial-level analysis

At the single-trial level, our goal was to dissociate the discrimination of kinematic information from the influence of top-down contextual factors, such as players strategic sophistication. Therefore, we separated trials by outcome and analysed only winning and losing trials. The underlying assumption was that if kinematic features enable reliable discrimination between hand poses before the pose is executed, this should influence readout accuracy and, consequently, the game outcome.

Movement variables were calculated only for wrist movements, as the RPS game is performed through up-and-down motion of the wrist, while finger configurations are revealed only shortly before the evaluation point (see Figure 1E; point E) Customized MATLAB code was created (Matlab; MathWorks Inc., Natick, MA) to calculate movement features from x-, y- and z-coordinates related to movement trajectory, temporal and kinematic features. A detailed explanation for each feature can be found in Table 1. All variables were calculated for each game and within the three different game phases (first swing, second swing, final swing; see Figure 1E and illustrated in Figure 3B) separately for each participant. We averaged the movement features for each game phase.^7^^,^^28^ The kinematic inference pipeline is illustrated in Figure 3A.

#### Kinematic encoding

To quantify how much intention information is encoded in single-trial kinematics, we fit a multinomial logistic regression that maps each trial’s feature vector *X* (see Table 1) to the categorical move label *Y* (rock, paper, scissors). The model yields class posteriors *P* for each trial and a predicted label $\hat{Y}=\arg\;\;\max\;P\text{.}$

Encoding accuracy is defined as the proportion of trials with $\hat{Y}=Y$. Confusion matrices are provided in the supplemental information Figure S1.

Beyond categorical accuracy, we quantified the intention information strength given the kinematic evidence for the true action choice using a log-odds margin. This measure reflects the separation between the true class and the strongest competitor assigned by the multinominal encoding model.

Formally, the encoding strength equals the difference in log-odds of the two most likely classes and therefore provides a graded measure of classification confidence: larger positive values indicate that the kinematics strongly supported the true action, whereas values near zero reflect ambiguous or weakly informative kinematics. In trials where the competing class is assigned higher probability than the true class, the measure becomes negative, indicating misclassification.

Importantly, intention labels were assigned post hoc based on the pose that was actually played, rather than being predefined or instructed in advance. This ensured that kinematic predictions were derived from naturally generated movements, preserving the dynamical and natural setting of the interaction.

#### Kinematic readout

To estimate likely kinematic readout, we modelled the extent to which an observer’s inferred expectation about the opponent’s action could be recovered from the opponent’s movement kinematics (Becchio et al., 2024; see Figure 3C). Whereas encoding maps an action intention onto movement, readout concerns the complementary mapping from observed movement onto an inferred action state. In the present paradigm, however, this inferred state was reconstructed from the observer’s behaviour rather than obtained from an explicit perceptual report. Accordingly, likely readout constitutes an indirect behavioural proxy rather than a direct measure of perceptual readout.

Under the task assumption that participants aimed to select the winning counteraction, each observed action *A*_*g*_ was mapped onto the opponent action it would defeat, providing an implicit estimate of the participant’s anticipated opponent move,

$$C_{g}=\text{inv}-\text{beat}\left(A_{g}\right)\text{,}$$

For example, choosing Rock corresponds to an inferred expectation that the opponent would play Scissors.

A separate multinomial logistic classifier was then trained to map the opponent’s single-trial kinematics $X_{g}^{\text{opp}}$ onto this inferred belief,

$$P_{g}\left(C=c|X_{g}^{\text{opp}}\right),\;c\in \left\{\text{Rock},\text{Paper},\text{Scissors}\right\}\text{,}$$

with the predicted belief defined as

$${\hat{C}}_{g}=\arg\max_{c}P_{g}\left(C=c|X_{g}^{\text{opp}}\right)\text{.}$$

This classifier was distinct from the encoding classifier: whereas encoding predicted the opponent’s actually executed action from their kinematics, likely readout predicted the observer’s choice-derived expectation from the opponent’s kinematics.

Likely-readout accuracy was defined as the proportion of games for which the model recovered the belief inferred from the participant’s action,

$${\hat{C}}_{g}=C_{g}\text{.}$$

Similar, to encoding strength we used log-odds margin to test whether, the model favoured the inferred action over its strongest competitor. This readout scalar represents the strength of kinematic evidence guiding the participants decision: positive values indicate games in which the participant acted in line with kinematic cues, while negative values denote games where behaviour contradicted the information encoded in the movement. Because explicit perceptual reports would have disrupted continuous competitive play, the present measure cannot establish participants’ perceptual beliefs directly. We therefore refer to it as likely kinematic readout throughout the manuscript to distinguish this choice-derived behavioural approximation from explicit perceptual readout.

#### Phase-dependent encoding and likely readout stability

To assess the temporal stability of information processing throughout the game, we quantified the consistency of both kinematic encoding and likely kinematic readout across the three movement phases (first, second, and final swing).

For **encoding stability**, a value of **1** (correct) was assigned for each phase when the pose predicted from that phase’s kinematics matched the true opponent pose,

$${\hat{Y}}_{\text{first}}^{\text{encoding}}=Y_{\text{first}},{\hat{Y}}_{\text{second}}^{\text{encoding}}=Y_{\text{second}},{\hat{Y}}_{\text{final}}^{\text{encoding}}=Y_{\text{final}}$$

The resulting three-element binary pattern (e.g., **111**) indexed whether the intended action was correctly encoded throughout the unfolding movement. For example, the pattern **011** indicates that decoding was incorrect during the first swing but correct during the second and final swings (Figure 2C).

For **likely readout stability**, an analogous binary measure was computed from the participant’s observed responses. A value of **1** (correct) was assigned for each phase when the participant selected the action implied by the likely kinematic readout, that is, the action that would optimally counter the opponent action predicted from that phase’s kinematics,

$${\hat{Y}}_{\text{first}}^{\text{readout}}=Y_{\text{first}}^{\text{likely}},{\hat{Y}}_{\text{second}}^{\text{readout}}=Y_{\text{second}}^{\text{likely}},{\hat{Y}}_{\text{final}}^{\text{readout}}=Y_{\text{final}}^{l\text{ikely}}$$

The resulting three-element pattern (e.g., **110**, Figure 2C) therefore reflects whether the participant consistently followed the likely kinematic readout across the unfolding movement.

Together, these complementary stability measures quantify how consistently intention information was represented in movement kinematics (encoding stability) and how consistently participants’ choices aligned with the likely kinematic readout over time.

#### Contextual inference of intentions

To model contextual inference, we compared four computational models that generate trial-wise posterior probabilities over the player’s next action from recent game history. The models fall into two conceptually distinct families. Action-based models predict choices from sequential regularities in previous actions, independent of their success, whereas outcome-based models adapt predictions according to the consequences of previous actions (wins, losses, or ties). This distinction allowed us to test whether successful performance is better explained by exploiting sequential action patterns or by reward-driven adaptation.

#### Action-based models: Markov model

The Markov model predicts the next action from first-order transition probabilities between consecutive actions. A 3\times3 transition matrix C was maintained, where each row corresponds to the previous action and each column to the subsequent action. The posterior probability of the next action was obtained from the smoothed transition counts,

$$P\left(Y_{g}|Y_{g-1}\right)=\frac{M\left(Y_{g-1},:\right)+0.5}{\sum_{j}M\left(Y_{g-1},j\right)+1.5}\text{,}$$

Using add -1/2 smoothing.

After each trial, the matrix was updated with exponential forgetting (*C*←*λC*) followed by incrementing the observed transition. Thus, predictions depended exclusively on sequential action statistics and were independent of trial outcomes.

#### Action-based models: Cognitive hierarchy assessment (CHASE)

The CHASE model formalizes recursive strategic reasoning by assuming that players differ in their level of strategic sophistication, denoted by k. A Bayesian posterior is maintained over three reasoning levels (k=0,1,2). At each game g, level-specific predictions are generated from the recent action history within a moving window of length h. The non-strategic level (k=0) predicts actions from the player’s own recent action frequencies, reflecting habitual responding. The first strategic level (k=1) predicts the action that best counters the opponent’s recent action frequencies, whereas the second strategic level (k=2) recursively best responds to the predicted k=1 strategy. The likelihood of the observed action is computed for each reasoning level, and the posterior over reasoning levels is updated recursively using Bayesian inference with exponential forgetting.

The resulting mixture distribution was obtained by marginalizing across reasoning levels,

$$P_{\text{mix}}\left(Y_{g}\right)=\sum_{k}P\left(Y_{g}|k\right)\;P\left(k|{\text{history}}_{1:g-1}\right)\text{,}$$

and was subsequently multiplied element-wise by an autoregressive prior over actions,

$$\pi_{g}(c)=\gamma_{\text{ar}}\;P\left(Y_{g-1}\right)>+\frac{1-\gamma_{\text{ar}}}{3}\text{,}$$

yielding the final posterior

$$P\left(Y_{g}\right)\propto P_{\text{mix}}\left(Y_{g}\right)\cdot \pi_{g}\text{,}$$

which was renormalized to sum to one. Here, *π*_*g*_ imposes mild temporal smoothing between consecutive predictions, with *γ*_ar_ controlling the strength of the autoregressive prior. To account for uncertainty in the inferred reasoning level, each level-specific action distribution was additionally shrunk toward the uniform distribution according to the entropy of the posterior over reasoning levels before computing the mixture. Model performance on each trial was quantified as the log-likelihood of the observed action under *P*(*Y*_*g*_), whereas the posterior probability assigned to the action actually played was used as the trial-wise posterior-strength measure in subsequent behavioural analyses.

#### Outcome-based models: Win-stay/lose-shift (WSLS)

The WSLS model predicts behaviour from the previous trial outcome by estimating the probability of repeating or rotating relative to the previous action. Outcome-specific transition matrices W were maintained separately for wins, losses, and ties. Given the previous outcome o, posterior probabilities were obtained from the smoothed row

$$P\left(r|o\right)=\frac{W\left(o,:\right)+0.5}{\sum_{j}W\left(o,j\right)+1.5}$$

where r denotes staying or rotating relative to the previous action. The observed relation was used to update the corresponding row with exponential forgetting after each trial. This formulation generalizes the classical Win–Stay/Lose–Shift strategy while allowing probabilistic deviations.

#### Outcome-based models: Reinforcement learning (RL)

The reinforcement-learning model implemented a Rescorla–Wagner learning rule with softmax action selection. Expected action values Q were updated after each trial according to

Q(a)←Q(a)+α[r−Q(a)]

where r was coded as 1 (win), 0.5 (tie), and 0 (loss). Trial-wise posterior probabilities were generated using a softmax policy,

$$P\left(Y_{g}=c\right)=\frac{\exp\left(\beta \;Q\left(c\right)\right)}{\sum_{j}\exp\left(\beta \;Q\left(j\right)\right)}\text{,}$$

where *β*controls choice stochasticity. Thus, predictions were driven by accumulated reward history rather than action sequences alone.

#### Encoding and readout accuracy

To test whether intention information was expressed in movement kinematics and differentially utilized by opponents as a function of game outcome and movement phase, encoding and readout were analysed at the single-trial level using hierarchical generalized linear mixed-effects models (GLMMs) with a binomial distribution and a logit link function. The underlying multinomial classifier was trained and evaluated separately for each participant using a within-participant three-fold cross-validation procedure. To preserve the temporal structure of the experiment, the sequence of 300 games was divided into three consecutive blocks. In each fold, two blocks were used for training and the remaining block for testing, such that each third of the session served once as the independent test set. Consequently, all decoding analyses were performed entirely within participants, and no information was shared across participants or dyads. Fixed effects included Outcome (win vs. loss), Game Phase (first swing, second swing, final swing), and their interaction. Random intercepts were included for participant and dyad to account for repeated observations and the nested structure of the data. Significant interactions were followed by estimated marginal means with multiplicity-adjusted pairwise comparisons. To assess whether encoding and readout exceeded chance performance, model-based estimates and their 95% confidence intervals were compared against the theoretical chance level of 1/3. Statistical significance was assessed at α = 0.05.

To assess the robustness and generalizability of the kinematic decoding approach, we conducted a series of supplementary control analyses, including cross-person decoding, early-phase decoding, permutation testing, and additional likely-readout sensitivity analyses (supplemental information Figures S2 and S3).

#### Statistical analysis of phase-stability scores

Winning and losing trials were compared on each score (weighted, binary, stability), separately for encoding and readout, using two-tailed independent-samples t-tests; group means, mean differences with 95% CIs, t(df), Cohen's d, and Holm–Bonferroni-corrected *p*-values (across the six comparisons) are reported. Because trials are nested within participants and dyads, each contrast was additionally confirmed with a linear mixed-effects model (score ∼ outcome + (1 | participant) + (1 | dyad)); the outcome coefficient (win − loss) is reported with its *p*-value. Draws were excluded. The categorical stability pattern (000–111) was compared between outcomes with a χ^2^ test.

#### Mixed-effects modelling of single-trial win probability

Single-trial win probability was analysed using binomial mixed-effects logistic regression with a logit link. Models were fitted by maximum likelihood using the Laplace approximation. Predictors comprised the posterior probabilities of the four contextual models—first-order Markov, win–stay/lose–shift (WSLS), reinforcement learning (RL), and CHASE—together with kinematic encoding strength and kinematic readout strength.

For each contextual model, posterior probability was defined as the probability assigned to the action actually selected by the player on trial g,

Sm,g=Pm(Yg=yg)

such that higher values indicate stronger support of the observed action by the respective model.

Kinematic encoding and readout strength were obtained from two cross-validated multinomial logistic classifiers, one for each channel, each using the same 33-dimensional joint kinematic representation of a trial. Specifically, the 11 kinematic features extracted from each of the three movement phases (first, second, and final swing) were concatenated into a single 33-dimensional feature vector, so that phase-specific information was integrated through the classifiers' learned regression weights rather than by averaging across phases. Encoding strength was defined as the posterior probability assigned to the player's own action, decoded from the player's own movement kinematics, whereas kinematic readout strength was defined as the posterior probability assigned to the opponent action implied by the player's observed countermove (i.e., the inferred belief underlying the chosen response), decoded from the opponent's movement kinematics. Thus, both kinematic measures constitute continuous probabilistic estimates derived from cross-validated classifiers.

All continuous predictors were standardized prior to analysis. Random intercepts for participant and dyad accounted for repeated observations and dependencies between interacting players. Analyses were restricted to decisive games, and the first game of each session was excluded because contextual models had not yet accumulated informative action or outcome history.

The unique contribution of each predictor was evaluated using likelihood-ratio tests comparing the full model with nested models in which the predictor of interest was omitted. All nested models were estimated using the same maximum-likelihood procedure, and Wald tests together with 95% confidence intervals are reported alongside the likelihood-ratio tests.

To evaluate robustness, three complementary analyses were performed. First, each contextual model was examined separately together with the two kinematic predictors. Second, the full model was refitted using within-participant centred predictors to isolate trial-to-trial variation from stable between-participant differences. Third, pairwise correlations and variance inflation factors were calculated to assess collinearity among the contextual predictors (see supplemental information Figure S4). All regression coefficients were interpreted as associative relationships with single-trial winning probability rather than as evidence for causal strategy use.

To visualize the relationship between posterior strength and behavioural success, trials were additionally grouped into quantiles of posterior strength separately for each model. Posterior strength was defined as the posterior probability assigned by a model to the action actually played on that trial. For each quantile, the observed winning probability was calculated and plotted against the corresponding posterior-strength level. These descriptive analyses were used to assess whether the relationship between posterior strength and winning was graded and monotonic rather than reflecting a binary high-versus-low effect. No inferential statistics were based on these visualizations; statistical inference relied on the mixed-effects logistic regression models described above.

#### Context–kinematic integration analysis

Before analysing situations of explicit disagreement between information sources, we first examined whether contextual and kinematic information each provided unique predictive information about single-trial winning and whether their contributions interacted. To quantify the unique contribution of each information source, nested likelihood-ratio tests first compared models containing only the four contextual predictors (Markov, WSLS, reinforcement learning, and CHASE) with models additionally including the two kinematic predictors (encoding strength and likely kinematic readout strength), and vice versa. Additional nested comparisons quantified the incremental contribution of each contextual predictor beyond the kinematic model, as well as the contribution of each kinematic predictor beyond each contextual model. Significant likelihood-ratio tests indicate that the added predictor(s) significantly improved predictive model fit.

To test whether contextual information modulated the influence of kinematic information on single-trial winning, the main-effects mixed-effects logistic regression model was extended by including interactions between the two kinematic predictors (encoding strength and likely kinematic readout strength) and each contextual model. The resulting model therefore contained the main effects of all six predictors together with eight kinematic–context interaction terms (encoding × context and likely readout × context for each contextual model). Interactions among contextual models and between encoding and likely kinematic readout were not included, as the aim was specifically to determine whether contextual information modulated the relationship between kinematic information and single-trial winning probability rather than modelling dependencies among the contextual models themselves.

All models were estimated by maximum likelihood using the Laplace approximation and included random intercepts for participant and dyad. Continuous predictors were z-standardized before interaction terms were computed. Evidence for context–kinematic integration was assessed by comparing the interaction model with the additive main-effects model using a likelihood-ratio test. A significant improvement in predictive model fit indicates that the contribution of kinematic information depends on the concurrent contextual state rather than contributing additively. Significant omnibus effects were followed by Wald tests of the individual interaction coefficients, with Holm correction applied across the eight interaction terms

#### Context–kinematic conflict analysis

To determine which source of information guided action selection when contextual and kinematic information recommended different actions, we performed a trial-wise conflict analysis. For each contextual model (Markov, WSLS, reinforcement learning, and CHASE), the contextual recommendation was defined as the action with the highest posterior probability,

$$c_{\text{context}}=\arg\;\;\max\;P_{\text{context}}\left(Y_{g}\right)\text{,}$$

whereas the kinematic recommendation corresponded to the action implied by the likely kinematic readout (i.e., the optimal counteraction given the decoded opponent intention).

The analysis was restricted to decisive games (wins/losses) in which the contextual and kinematic models recommended different actions. For a robustness check including draws see supplemental information Figure S5. Trials on which both models recommended the same action were excluded because these trials do not allow successful action selection to be attributed to one information source rather than the other. Likewise, trials in which participants selected neither recommended action were omitted.

For the remaining trials, behaviour was classified according to whether the participant followed the contextual recommendation or the likely kinematic readout. Separate mixed-effects logistic regression models were fitted for each contextual model, predicting single-trial outcome while including random intercepts for participant and dyad. Models were estimated by maximum likelihood using the Laplace approximation. Odds ratios greater than one indicate that following the contextual recommendation increased the odds of winning relative to following the likely kinematic readout under conditions of direct disagreement.

To compare the relative advantage of the contextual models, participant-wise winning rates were additionally calculated separately for context-guided and readout-guided decisions. For each participant, the advantage of a contextual model was defined as the difference in winning probability between context-guided and readout-guided trials. Differences between contextual models were evaluated using paired Wilcoxon signed-rank tests with Holm correction for multiple comparisons.
