# Supplementary material for: Context shapes action understanding in social interactions
Source: iScience. 2026 Oct 9;29(11):117796. doi: 10.1016/j.isci.2026.117796 (PMC13677244; doi:10.1016/j.isci.2026.117796)
Supplement: Supplementary file 1 [file mmc1.pdf]

iScience, Volume *29*

Supplemental information

Context shapes action understanding in social interactions

Johannes Keck, Kazuyuki Sato, and Rouwen Cañal-Bruland

Supplementary Materials

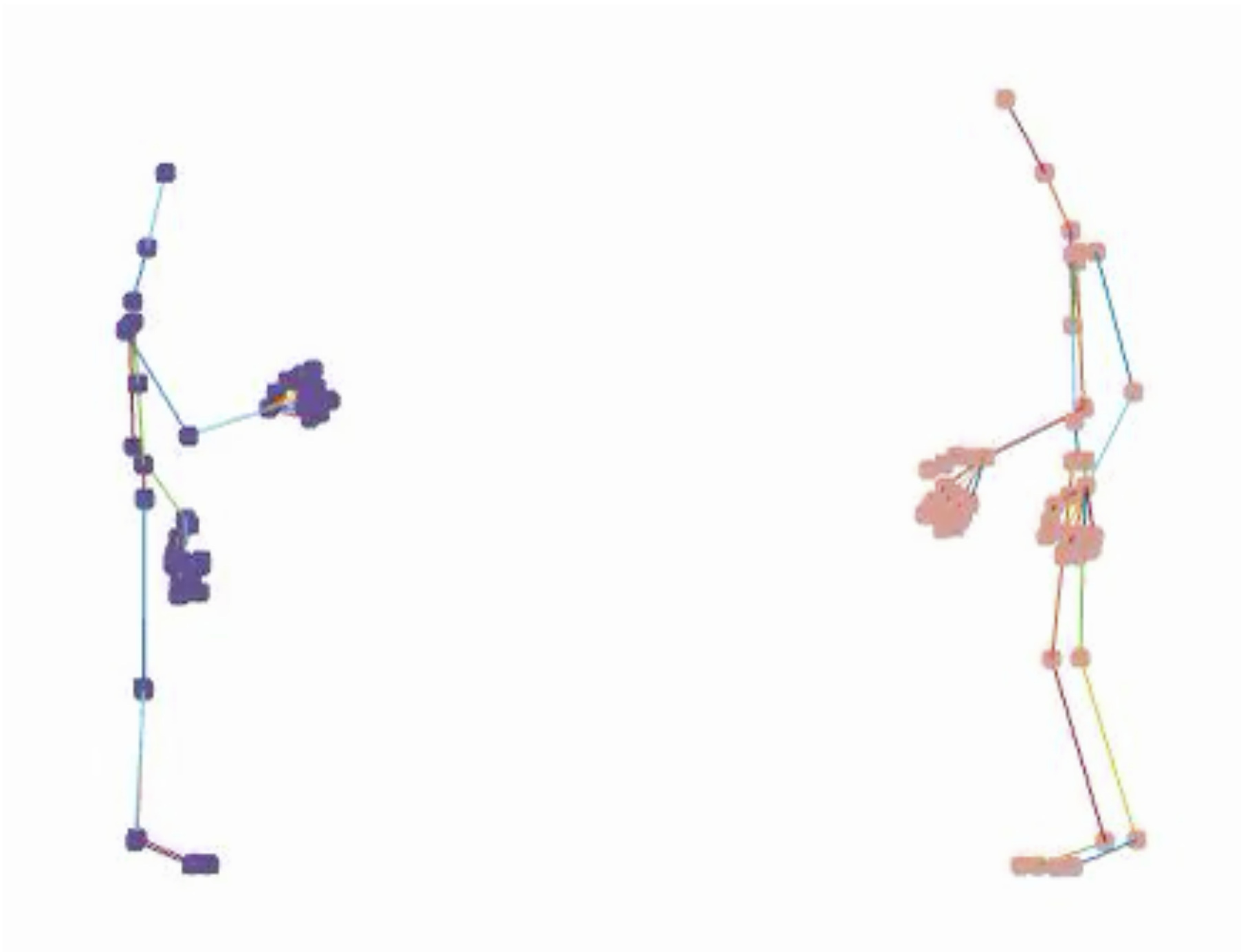

Supplementary Material Video 1 shows a representative rock-paper-scissors interaction with motion caption markers (rps_full_body_example.mp4) and can be found in the OSF repository https://doi.org/10.17605/OSF.IO/89XS5.

Dyad	**Participant 1**	**Participant 2**
10	NOT FINISHED	
20	Unreliable handshape reconstruction	
25	Unreliable handshape reconstruction	
32		Unreliable handshape reconstruction
37	NOT FINISHED	
39	Unreliable handshape reconstruction	
43	Unreliable handshape reconstruction	

Supplementary Methods Table 1. Exclusion Criteria

Supplementary Results

Kinematic	Game phase	Group	Accuracy	95 % CI
Encoding	First	Win	0.428	[0.409, 0.447]
		Loss	0.423	[0.404, 0.442]
	Second	Win	0.428	[0.409, 0.447]
		Loss	0.431	[0.412, 0.450]
	Final	Win	0.428	[0.409, 0.447]
		Loss	0.418	[0.399, 0.437]
Likely readout	First	Win	0.338	[0.316, 0.360]
		Loss	0.377	[0.354, 0.400]
	Second	Win	0.333	[0.311, 0.355]
		Loss	0.382	[0.359, 0.405]
	Final	Win	0.311	[0.290, 0.333]
		Loss	0.382	[0.359, 0.406]

Supplementary Results Table 1: Descriptive statistics for encoding and readout across all games and players.

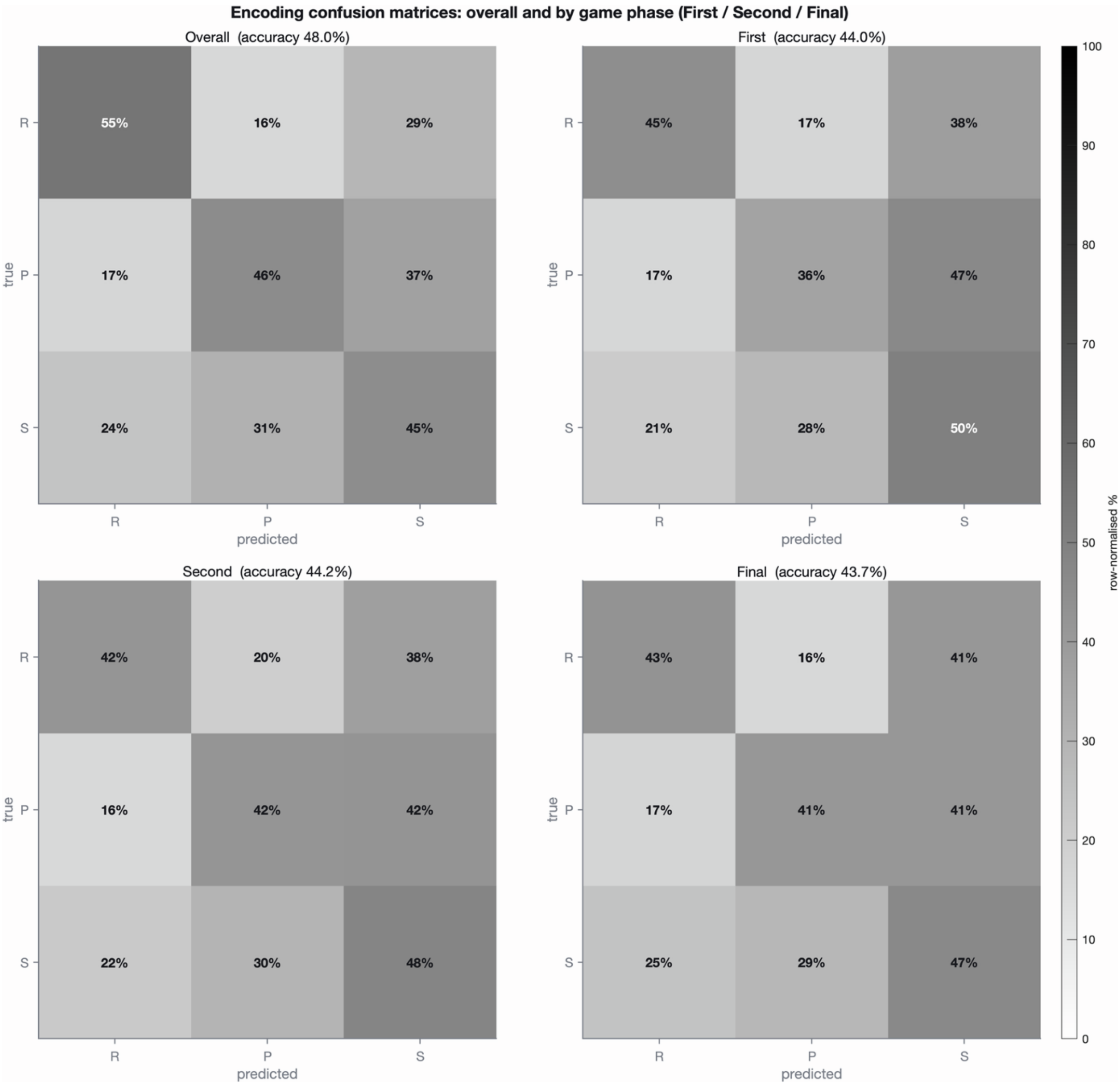

Supplementary Results Figure 1: Encoding performance across game phases. Average confusion matrices of the kinematic encoding model across all sessions and participants for the three game phases (first, secpmd, and final swing) and the overall performance. Rows represent the true hand shape (rock, paper, scissors), and columns represent the predicted class from the multinomial logistic regression model based on kinematic features. Each cell shows the mean number of trials classified into a given category across sessions and players, with the standard deviation indicated below (mean ± SD). Darker shading indicates higher counts. Overall encoding accuracy across all phases is reported above the panels, and phase-specific accuracies are shown in each subplot title. Confusion matrices are aggregated across both players and sessions.

Encoding Player x Game Phase

session	player	enc_1st_mean	enc_1st_sd	enc_2nd_mean	enc_2nd_sd	enc_final_mean	enc_final_sd
**Dyad_11**	player_one	0.36	0.09	0.36	0.09	0.37	0.08
**Dyad_11**	player_two	0.35	0.08	0.35	0.08	0.36	0.08
**Dyad_12**	player_one	0.36	0.09	0.37	0.08	0.37	0.09
**Dyad_12**	player_two	0.38	0.13	0.37	0.13	0.38	0.12
**Dyad_13**	player_one	0.36	0.08	0.37	0.08	0.37	0.08
**Dyad_13**	player_two	0.38	0.1	0.37	0.1	0.37	0.08
**Dyad_14**	player_one	0.36	0.07	0.37	0.08	0.36	0.08
**Dyad_14**	player_two	0.36	0.07	0.36	0.09	0.38	0.09
**Dyad_15**	player_one	0.35	0.08	0.35	0.08	0.36	0.09
**Dyad_15**	player_two	0.35	0.08	0.35	0.07	0.34	0.09
**Dyad_16**	player_one	0.43	0.2	0.41	0.18	0.4	0.17
**Dyad_16**	player_two	0.39	0.21	0.36	0.17	0.37	0.16
**Dyad_17**	player_one	0.35	0.11	0.35	0.1	0.35	0.1
**Dyad_17**	player_two	0.39	0.1	0.39	0.09	0.37	0.08
**Dyad_18**	player_one	0.38	0.1	0.4	0.11	0.38	0.1
**Dyad_18**	player_two	0.35	0.11	0.34	0.13	0.36	0.09
**Dyad_19**	player_one	0.36	0.05	0.35	0.06	0.35	0.08
**Dyad_19**	player_two	0.37	0.06	0.37	0.08	0.38	0.08
**Dyad_1**	player_one	0.35	0.06	0.36	0.07	0.35	0.1
**Dyad_1**	player_two	0.34	0.07	0.34	0.08	0.35	0.12
**Dyad_21**	player_one	0.35	0.07	0.36	0.07	0.37	0.08
**Dyad_21**	player_two	0.35	0.09	0.35	0.09	0.37	0.08
**Dyad_22**	player_one	0.38	0.07	0.37	0.08	0.37	0.07
**Dyad_22**	player_two	0.38	0.08	0.37	0.09	0.38	0.08
**Dyad_23**	player_one	0.35	0.07	0.36	0.08	0.36	0.08
**Dyad_23**	player_two	0.36	0.06	0.37	0.07	0.36	0.11
**Dyad_24**	player_one	0.36	0.08	0.36	0.09	0.37	0.09
**Dyad_24**	player_two	0.37	0.09	0.36	0.09	0.39	0.09
**Dyad_26**	player_one	0.34	0.06	0.34	0.06	0.32	0.07
**Dyad_26**	player_two	0.34	0.07	0.34	0.07	0.35	0.06
**Dyad_27**	player_one	0.36	0.09	0.37	0.1	0.35	0.1
**Dyad_27**	player_two	0.35	0.07	0.35	0.07	0.35	0.08
**Dyad_28**	player_one	0.36	0.07	0.36	0.07	0.36	0.07
**Dyad_28**	player_two	0.34	0.06	0.34	0.06	0.35	0.08
**Dyad_29**	player_one	0.34	0.08	0.33	0.08	0.32	0.09
**Dyad_29**	player_two	0.34	0.08	0.34	0.09	0.37	0.07
**Dyad_2**	player_one	0.37	0.12	0.38	0.11	0.37	0.13
**Dyad_2**	player_two	0.34	0.05	0.35	0.06	0.38	0.07
**Dyad_30**	player_one	0.37	0.09	0.37	0.09	0.36	0.08
**Dyad_30**	player_two	0.37	0.11	0.35	0.1	0.35	0.07
**Dyad_31**	player_one	0.37	0.1	0.36	0.08	0.36	0.11
**Dyad_31**	player_two	0.37	0.12	0.36	0.11	0.38	0.12
**Dyad_33**	player_one	0.36	0.1	0.38	0.09	0.37	0.1

**Dyad_33**	player_two	0.36	0.08	0.36	0.09	0.38	0.11
**Dyad_34**	player_one	0.38	0.08	0.37	0.08	0.38	0.07
**Dyad_34**	player_two	0.39	0.09	0.39	0.09	0.39	0.09
**Dyad_35**	player_one	0.37	0.09	0.36	0.09	0.37	0.07
**Dyad_35**	player_two	0.41	0.08	0.4	0.09	0.4	0.09
**Dyad_36**	player_one	0.4	0.12	0.37	0.13	0.37	0.09
**Dyad_36**	player_two	0.36	0.1	0.34	0.09	0.35	0.1
**Dyad_38**	player_one	0.37	0.13	0.38	0.12	0.39	0.13
**Dyad_38**	player_two	0.39	0.09	0.39	0.1	0.37	0.1
**Dyad_3**	player_one	0.36	0.08	0.37	0.1	0.35	0.12
**Dyad_3**	player_two	0.35	0.09	0.36	0.1	0.36	0.1
**Dyad_40**	player_one	0.33	0.15	0.33	0.13	0.34	0.1
**Dyad_40**	player_two	0.34	0.06	0.35	0.06	0.35	0.07
**Dyad_41**	player_one	0.36	0.06	0.35	0.07	0.34	0.07
**Dyad_41**	player_two	0.37	0.08	0.37	0.08	0.37	0.07
**Dyad_42**	player_one	0.38	0.14	0.38	0.13	0.38	0.13
**Dyad_42**	player_two	0.38	0.11	0.38	0.1	0.36	0.06
**Dyad_44**	player_one	0.4	0.08	0.39	0.09	0.4	0.1
**Dyad_44**	player_two	0.39	0.1	0.39	0.1	0.37	0.12
**Dyad_45**	player_one	0.38	0.14	0.38	0.14	0.39	0.15
**Dyad_45**	player_two	0.39	0.11	0.38	0.11	0.34	0.08
**Dyad_4**	player_one	0.38	0.1	0.37	0.09	0.41	0.1
**Dyad_4**	player_two	0.39	0.07	0.39	0.07	0.38	0.06
**Dyad_5**	player_one	0.38	0.08	0.38	0.09	0.37	0.08
**Dyad_5**	player_two	0.39	0.09	0.38	0.09	0.38	0.07
**Dyad_6**	player_one	0.36	0.08	0.38	0.08	0.36	0.09
**Dyad_6**	player_two	0.34	0.07	0.35	0.07	0.35	0.08
**Dyad_7**	player_one	0.37	0.08	0.38	0.08	0.37	0.08
**Dyad_7**	player_two	0.37	0.08	0.38	0.08	0.38	0.08
**Dyad_8**	player_one	0.37	0.08	0.36	0.08	0.37	0.06
**Dyad_8**	player_two	0.36	0.07	0.37	0.07	0.37	0.1
**Dyad_9**	player_one	0.38	0.08	0.38	0.08	0.38	0.07
**Dyad_9**	player_two	0.37	0.06	0.37	0.06	0.38	0.07

Supplementary Results Table 2:. Encoding confidence across game phases and participants. Mean encoding confidence (± SD) for each participant across the three game phases (AB, BC, and CE). Encoding confidence reflects the posterior probability that the multinomial logistic regression model assigns to the true hand shape based on kinematic features. Values are averaged across trials within each session and player. Higher values indicate stronger kinematic evidence supporting the correct hand-shape classification.

Readout Player x Game Phase

session	player	rout_1st_mean	rout _1st_sd	rout_2nd_mean	rout _2nd_sd	rout _final_mean	rout _final_sd
**Dyad_11**	player_one	0.33	0.04	0.33	0.04	0.33	0.04

**Dyad_11**	player_two	0.34	0.04	0.34	0.04	0.34	0.04
**Dyad_12**	player_one	0.33	0.06	0.33	0.06	0.33	0.06
**Dyad_12**	player_two	0.34	0.05	0.34	0.05	0.34	0.05
**Dyad_13**	player_one	0.33	0.04	0.33	0.04	0.33	0.04
**Dyad_13**	player_two	0.33	0.04	0.33	0.04	0.33	0.05
**Dyad_14**	player_one	0.34	0.05	0.34	0.05	0.34	0.05
**Dyad_14**	player_two	0.33	0.04	0.34	0.04	0.34	0.04
**Dyad_15**	player_one	0.33	0.04	0.33	0.04	0.33	0.04
**Dyad_15**	player_two	0.33	0.04	0.33	0.04	0.33	0.04
**Dyad_16**	player_one	0.37	0.09	0.37	0.09	0.37	0.11
**Dyad_16**	player_two	0.37	0.1	0.38	0.1	0.38	0.12
**Dyad_17**	player_one	0.34	0.06	0.34	0.06	0.34	0.04
**Dyad_17**	player_two	0.36	0.07	0.36	0.07	0.36	0.05
**Dyad_18**	player_one	0.34	0.06	0.34	0.07	0.34	0.04
**Dyad_18**	player_two	0.35	0.06	0.35	0.06	0.34	0.04
**Dyad_19**	player_one	0.33	0.04	0.33	0.04	0.33	0.04
**Dyad_19**	player_two	0.34	0.04	0.34	0.04	0.34	0.04
**Dyad_1**	player_one	0.34	0.04	0.34	0.04	0.34	0.04
**Dyad_1**	player_two	0.33	0.04	0.33	0.05	0.33	0.04
**Dyad_21**	player_one	0.34	0.05	0.34	0.05	0.35	0.04
**Dyad_21**	player_two	0.35	0.05	0.35	0.05	0.35	0.05
**Dyad_22**	player_one	0.33	0.04	0.33	0.04	0.33	0.04
**Dyad_22**	player_two	0.33	0.03	0.32	0.03	0.33	0.04
**Dyad_23**	player_one	0.35	0.05	0.35	0.04	0.34	0.04
**Dyad_23**	player_two	0.34	0.04	0.34	0.05	0.34	0.04
**Dyad_24**	player_one	0.35	0.05	0.34	0.04	0.34	0.05
**Dyad_24**	player_two	0.34	0.04	0.34	0.04	0.34	0.04
**Dyad_26**	player_one	0.33	0.04	0.33	0.04	0.33	0.04
**Dyad_26**	player_two	0.35	0.04	0.35	0.05	0.34	0.04
**Dyad_27**	player_one	0.34	0.04	0.34	0.04	0.34	0.04
**Dyad_27**	player_two	0.34	0.06	0.34	0.06	0.34	0.06
**Dyad_28**	player_one	0.33	0.04	0.34	0.04	0.34	0.04
**Dyad_28**	player_two	0.34	0.04	0.33	0.04	0.33	0.04
**Dyad_29**	player_one	0.34	0.04	0.34	0.04	0.34	0.04
**Dyad_29**	player_two	0.34	0.05	0.35	0.05	0.35	0.04
**Dyad_2**	player_one	0.33	0.04	0.34	0.04	0.33	0.04
**Dyad_2**	player_two	0.34	0.07	0.34	0.06	0.34	0.06
**Dyad_30**	player_one	0.35	0.04	0.35	0.04	0.35	0.04
**Dyad_30**	player_two	0.34	0.05	0.33	0.05	0.34	0.05
**Dyad_31**	player_one	0.36	0.06	0.36	0.05	0.35	0.05
**Dyad_31**	player_two	0.33	0.05	0.33	0.05	0.33	0.05
**Dyad_33**	player_one	0.34	0.04	0.34	0.04	0.34	0.04
**Dyad_33**	player_two	0.33	0.04	0.33	0.04	0.34	0.04
**Dyad_34**	player_one	0.34	0.04	0.34	0.04	0.33	0.05
**Dyad_34**	player_two	0.35	0.03	0.35	0.03	0.36	0.04

**Dyad_35**	player_one	0.34	0.04	0.34	0.04	0.34	0.05
**Dyad_35**	player_two	0.34	0.05	0.34	0.05	0.34	0.05
**Dyad_36**	player_one	0.33	0.05	0.34	0.05	0.33	0.05
**Dyad_36**	player_two	0.33	0.05	0.33	0.06	0.33	0.04
**Dyad_38**	player_one	0.34	0.05	0.34	0.05	0.34	0.05
**Dyad_38**	player_two	0.35	0.04	0.35	0.04	0.35	0.04
**Dyad_3**	player_one	0.33	0.04	0.33	0.04	0.33	0.05
**Dyad_3**	player_two	0.33	0.05	0.33	0.05	0.32	0.05
**Dyad_40**	player_one	0.33	0.04	0.33	0.04	0.33	0.04
**Dyad_40**	player_two	0.34	0.07	0.34	0.07	0.34	0.05
**Dyad_41**	player_one	0.34	0.04	0.34	0.04	0.34	0.04
**Dyad_41**	player_two	0.34	0.04	0.34	0.04	0.34	0.04
**Dyad_42**	player_one	0.35	0.05	0.35	0.05	0.34	0.04
**Dyad_42**	player_two	0.38	0.07	0.37	0.06	0.35	0.06
**Dyad_44**	player_one	0.34	0.05	0.34	0.05	0.34	0.05
**Dyad_44**	player_two	0.35	0.05	0.35	0.05	0.34	0.04
**Dyad_45**	player_one	0.34	0.04	0.34	0.04	0.34	0.04
**Dyad_45**	player_two	0.34	0.06	0.34	0.06	0.34	0.07
**Dyad_4**	player_one	0.33	0.04	0.34	0.04	0.34	0.04
**Dyad_4**	player_two	0.34	0.05	0.34	0.04	0.34	0.05
**Dyad_5**	player_one	0.34	0.06	0.34	0.06	0.34	0.05
**Dyad_5**	player_two	0.36	0.04	0.37	0.04	0.36	0.04
**Dyad_6**	player_one	0.34	0.04	0.34	0.05	0.33	0.03
**Dyad_6**	player_two	0.34	0.05	0.33	0.05	0.34	0.04
**Dyad_7**	player_one	0.33	0.04	0.34	0.04	0.34	0.04
**Dyad_7**	player_two	0.35	0.05	0.36	0.05	0.36	0.05
**Dyad_8**	player_one	0.35	0.04	0.35	0.04	0.35	0.05
**Dyad_8**	player_two	0.33	0.05	0.33	0.05	0.33	0.04
**Dyad_9**	player_one	0.34	0.04	0.34	0.04	0.34	0.04
**Dyad_9**	player_two	0.34	0.04	0.33	0.04	0.34	0.05

Supplementary Results Table 3: Readout accuracy across game phases and participants. Mean readout accuracy (± SD) for each participant across the three game phases (AB, BC, and CE). Readout accuracy indicates whether the responder selected the move that would optimally counter the opponent's move predicted from kinematic information. Values are averaged across trials within each session and player. Higher values reflect more successful use of kinematic information to understand the opponent's action.

Pattern	Winning Total	Winning %	Losing Total	Losing %	Total
000	3089	52.1	2839	47.9	5928
001	348	49.2	360	50.8	708
010	209	52.1	192	47.9	401
011	186	47.1	209	52.9	395

100	244	56.0	192	44.0	436
101	180	49.2	186	50.8	366
110	441	55.9	348	44.1	789
111	1109	42.8	1481	57.2	2590
	**5806**		**5806**		**11612**

Supplementary Results Table 4. Frequency of temporal readout patterns across winning and losing trials. Readout patterns across the three game phases (AB, BC, and CE) are shown separately for winning and losing trials. Each pattern indicates whether readout was incorrect (0) or correct (1) in a given phase, yielding eight possible temporal sequences from 000 to 111. "Winning Total" and "Losing Total" denote the number of trials showing each pattern, and percentages are calculated within each outcome category. The final row reports the total number of winning and losing trials included in the analysis. This table complements the stability analysis by showing how specific temporal readout trajectories were distributed across trial outcomes.

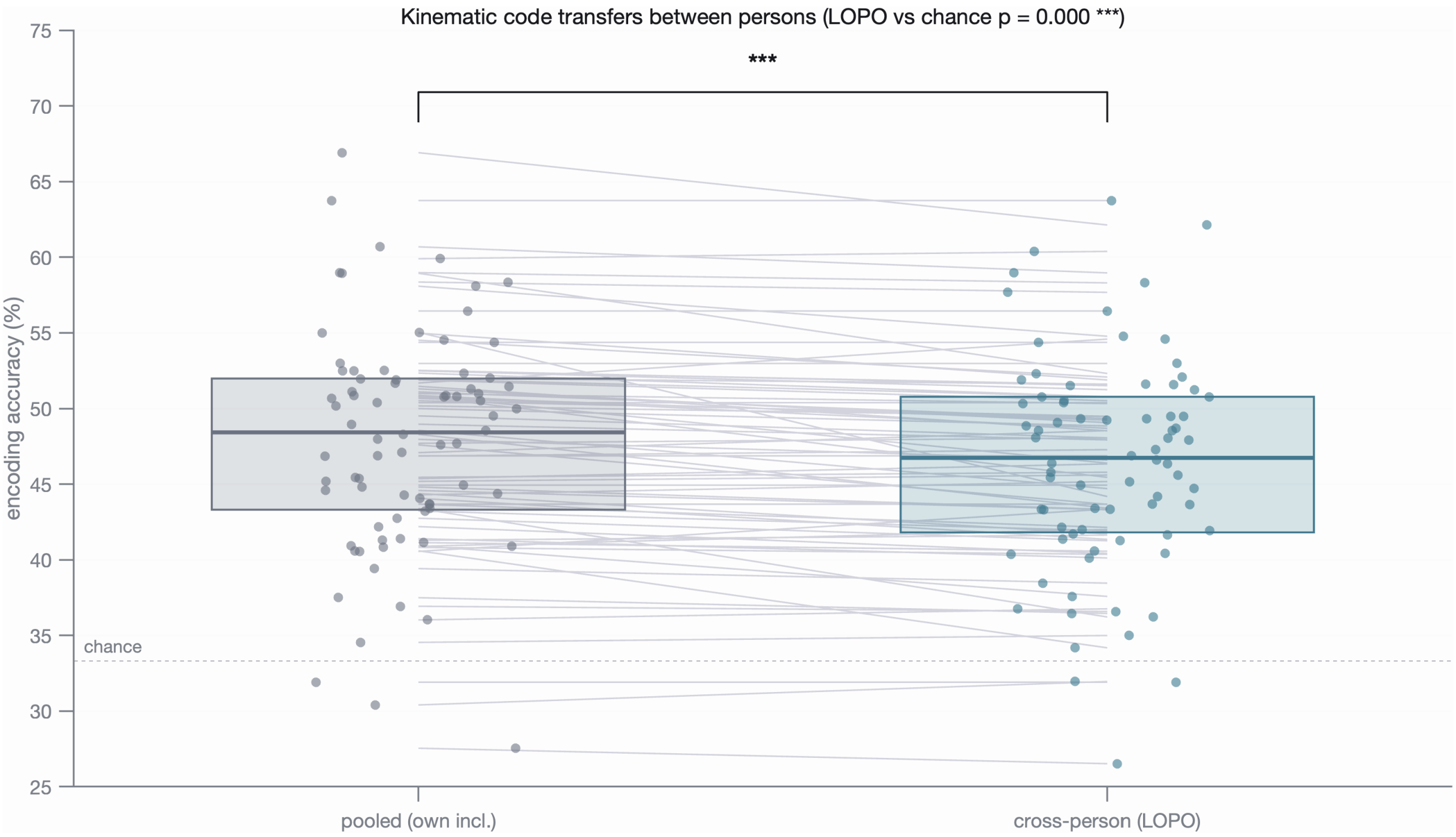

Supplementary Results Figure 2. Kinematic code transfers between participants. Encoding accuracy when the classifier was trained with the participant's own trials included (pooled) versus trained exclusively on all other participants and tested on the held-out person (cross-person, leave-one-person-out); grey lines connect the same participant across conditions, boxes show median and interquartile range. Cross-person decoding remained well above chance (dashed line = 33.3 %; Wilcoxon signed-rank vs chance, $p < .001$) and only marginally below the pooled estimate ($p < .001$), indicating that the kinematic code specifying an action is largely shared across individuals rather than idiosyncratic.

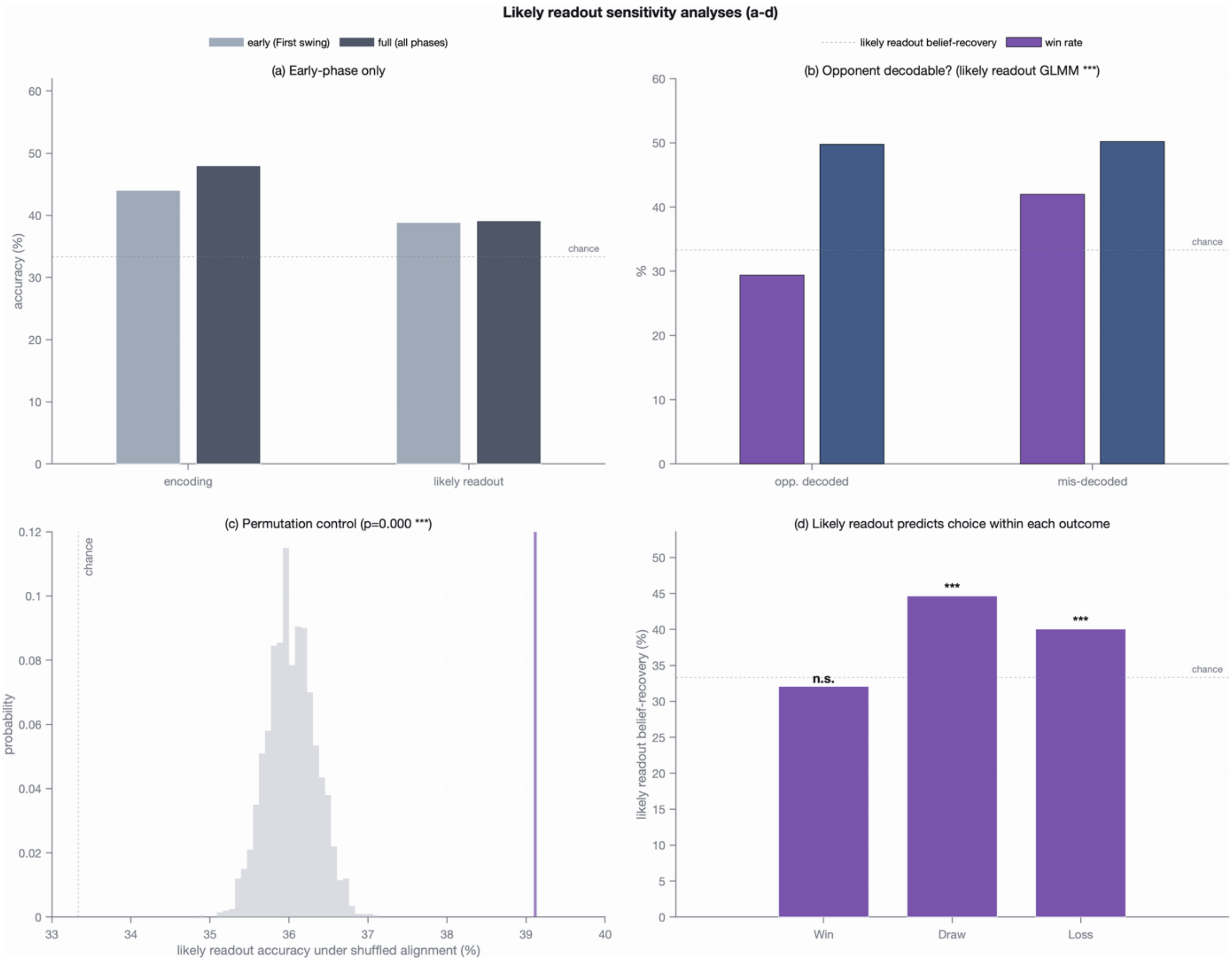

Supplementary Results Figure 3. Likely-readout sensitivity analyses. a) Encoding and likely-readout accuracy using only the first movement phase versus all phases; both exceeded chance already from the first swing, indicating that decoding reflects anticipatory information rather than the near-complete gesture. (b) Likely-readout belief-recovery (purple) and win rate (blue) split by whether the opponent's action was correctly decoded by the encoding model; readout accuracy depended on opponent decodability (mixed-effects GLMM,), whereas win rate did not. (c) Observed likely-readout accuracy (vertical line) relative to a shuffled-alignment permutation null (2,000 permutations, grey), confirming that the readout exceeded chance-level model–choice alignment ($p < .001$). (d) Likely-readout belief-recovery within each outcome: above chance on draws and losses (**) but at chance on wins (n.s.), showing that reading was informative about choices specifically on non-winning trials. Dashed lines mark chance (33.3 %).

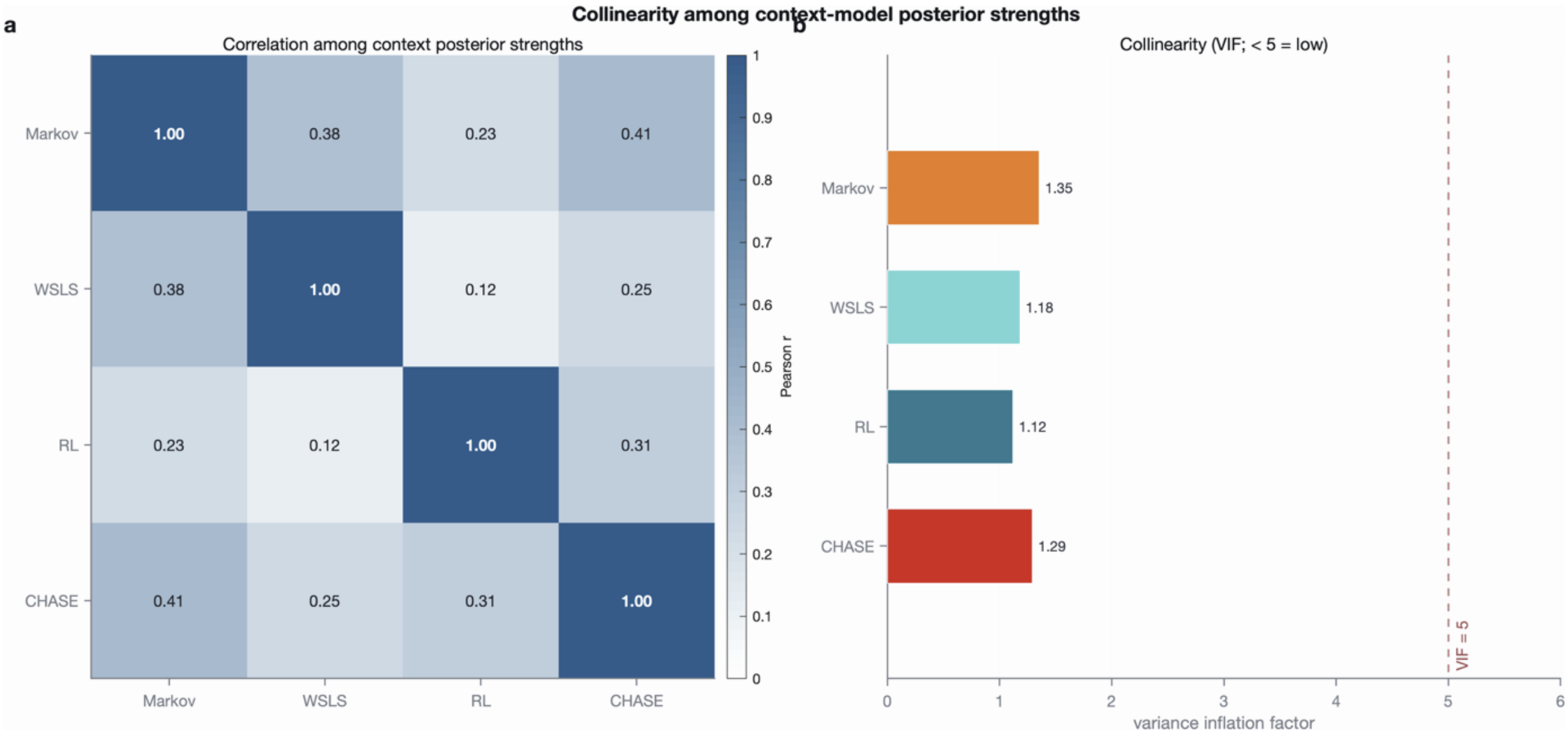

Supplementary Results Figure 4. Collinearity among context-model posterior strengths. (a) Pearson correlations among the four context-model posterior strengths (all $r \leq 0.41$). (b) Variance inflation factors for each predictor in the joint model (all ≤ 1.35, well below the conventional threshold of 5, dashed line). Together these indicate that the context predictors carried largely non-redundant information, supporting the stability of the joint-model estimates.

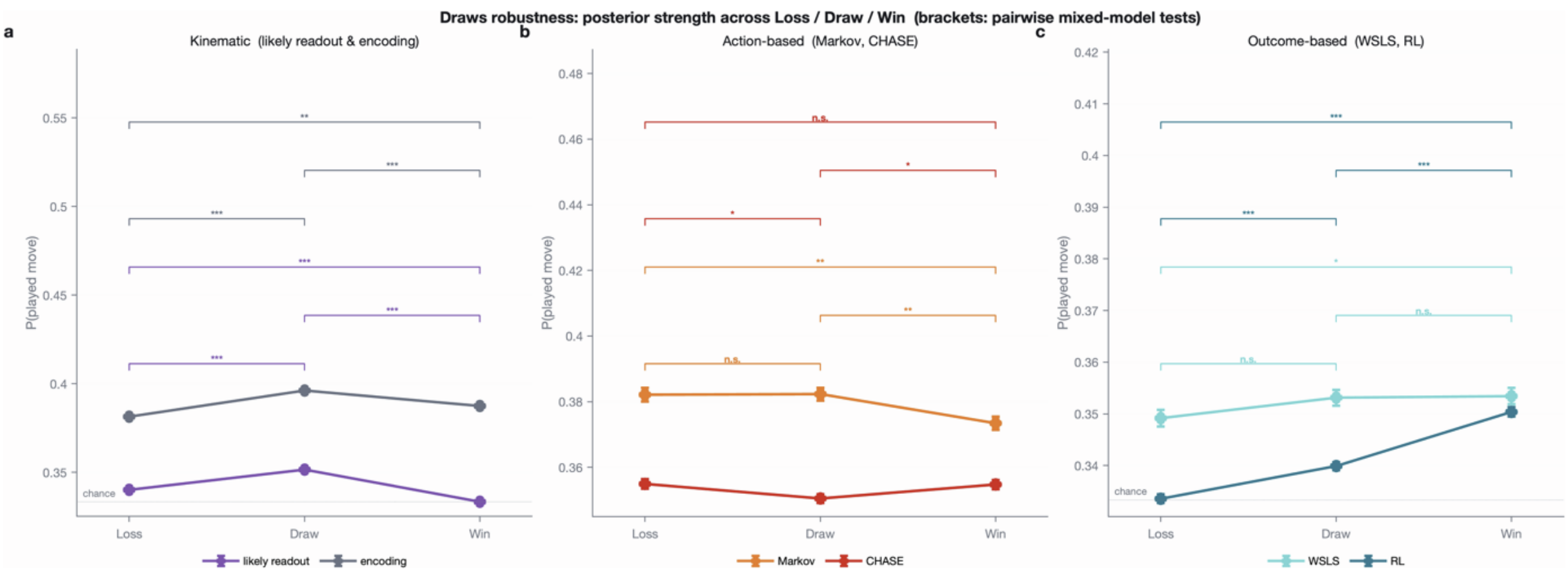

Supplementary Results Figure 5. Draw robustness. Model-estimated posterior strength assigned to the played move (mean ± SEM) across the ordered outcomes Loss < Draw < Win, with draws retained as a neutral intermediate category; brackets denote pairwise linear-mixed-model contrasts. (a) Kinematic predictors: likely-readout strength peaked on draws and was lowest on wins, whereas encoding was near-flat. (b) Action-based context models (Markov, CHASE). (c) Outcome-based context models: RL increased monotonically from losses through draws to wins, whereas WSLS showed a weaker rise. The graded pattern through the neutral draw category confirms a genuine dose–response relationship rather than a binary win–loss artefact. Dashed line = chance (1/3). *p < .05, **p < .01, ***p < .001.
